# Redox-Driven Antiaromaticity Enables Multistate Conductance in 1D Topological Insulators

**DOI:** 10.1021/jacs.6c15721

**Published:** 2026-09-07

**Authors:** Woojung Lee, Wanzhuo Shi, Junho Kwon, Jonathan Low, Sujun Wei, Xiaodong Yin, Luis M. Campos, Latha Venkataraman

**Affiliations:** † Department of Chemistry, 5798 Columbia University, New York, New York 10027, United States; ‡ Institute of Science and Technology Austria, Am Campus 1, Klosterneuburg 3400, Austria; § Department of Chemistry, Queensborough Community College of the City, University of New York, Bayside, New York 11364, United States; ∥ Department of Applied Physics and Applied Mathematics, Columbia University, New York, New York 10027, United States

## Abstract

Conjugated molecules with diradical character have emerged as promising building blocks for high-conductance single-molecule electronics because of their behavior as 1D topological insulators. However, the impact of antiaromaticity on these radical-based edge states has not been explored. Here, we introduce thiophene-based molecular wires that exhibit intrinsic diradical character in their ground state under ambient conditions and systematically investigate how their conductance responds to changes in aromaticity upon the oxidation of fluorene-based flanking moieties. Using scanning tunneling microscopy-based break junction (STM-BJ) techniques, we show that the conductance increases with molecular length in the neutral state, supporting one-dimensional topological behavior. We further examine the effect of oxidation on conductance through three complementary methods, revealing substantial conductance enhancements that we attribute to an increase in the antiaromatic character of the wires. Together, these findings establish a rational design strategy for highly conducting, electrochemically switchable molecular junctions, with implications for single-molecule electronics.

## Graphical abstract

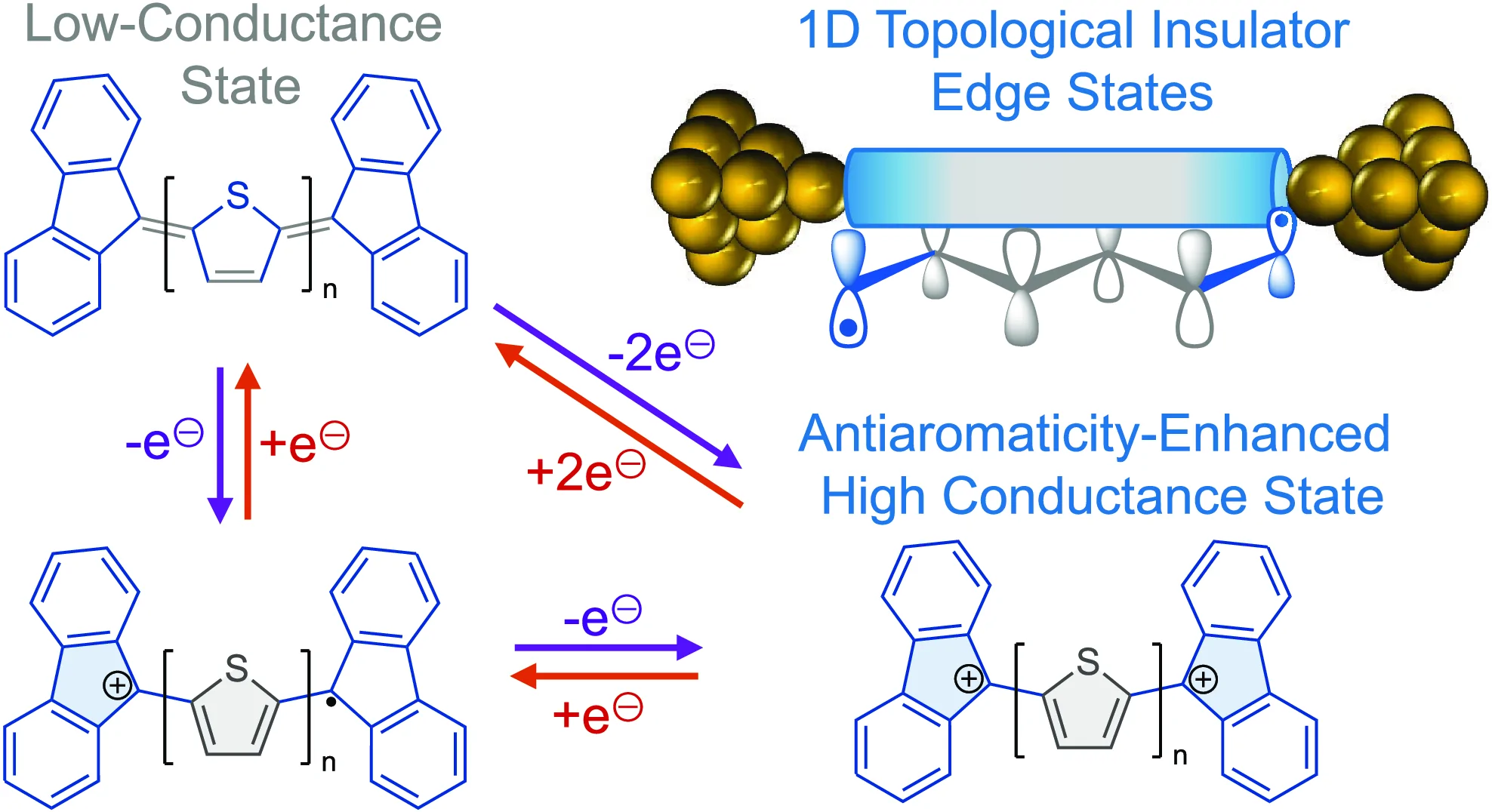

## Introduction

Over the last two decades, molecular analogs of electronic devices with a wide range of functionalities have been creatively developed.^1–3^ Significant effort is generally focused on controlling electron transport through metal–molecule–metal junctions by tuning chemical functionality and connectivity of conjugated organic molecules.^4–10^ Modulating electronic properties through molecular engineering allows controlling transport through π-bonds, which is more effective than σ-bonds, especially in long molecules.^11–14^ In many cases, structure–property relationships have been studied connecting physicochemical principles using single-molecule conductance measurements. Recent interests are now turning toward understanding the role of orbital topology—the adaptation of the Su, Schrieffer, Heeger (SSH)^15^ polyacetylene soliton model for the development of molecular one-dimensional topological insulators (1D-TIs, Figure 1a).^16^ To date, single molecules bearing one-electron p-orbitals on nitrogen and carbon centers have been found to give rise to unusually high conductance as a function of increasing molecular length.^17^ Such behavior is a hallmark of 1D-TIs, akin to two-dimensional systems with a charge-insulating interior and whose dissipationless edge states support ballistic charge transport.^18^ The resulting reversed conductance decay trend of 1D-TIs has been both theoretically and experimentally validated, enabling the design of long, highly conductive molecular wires.^10,17,19–26^

**Figure 1:**
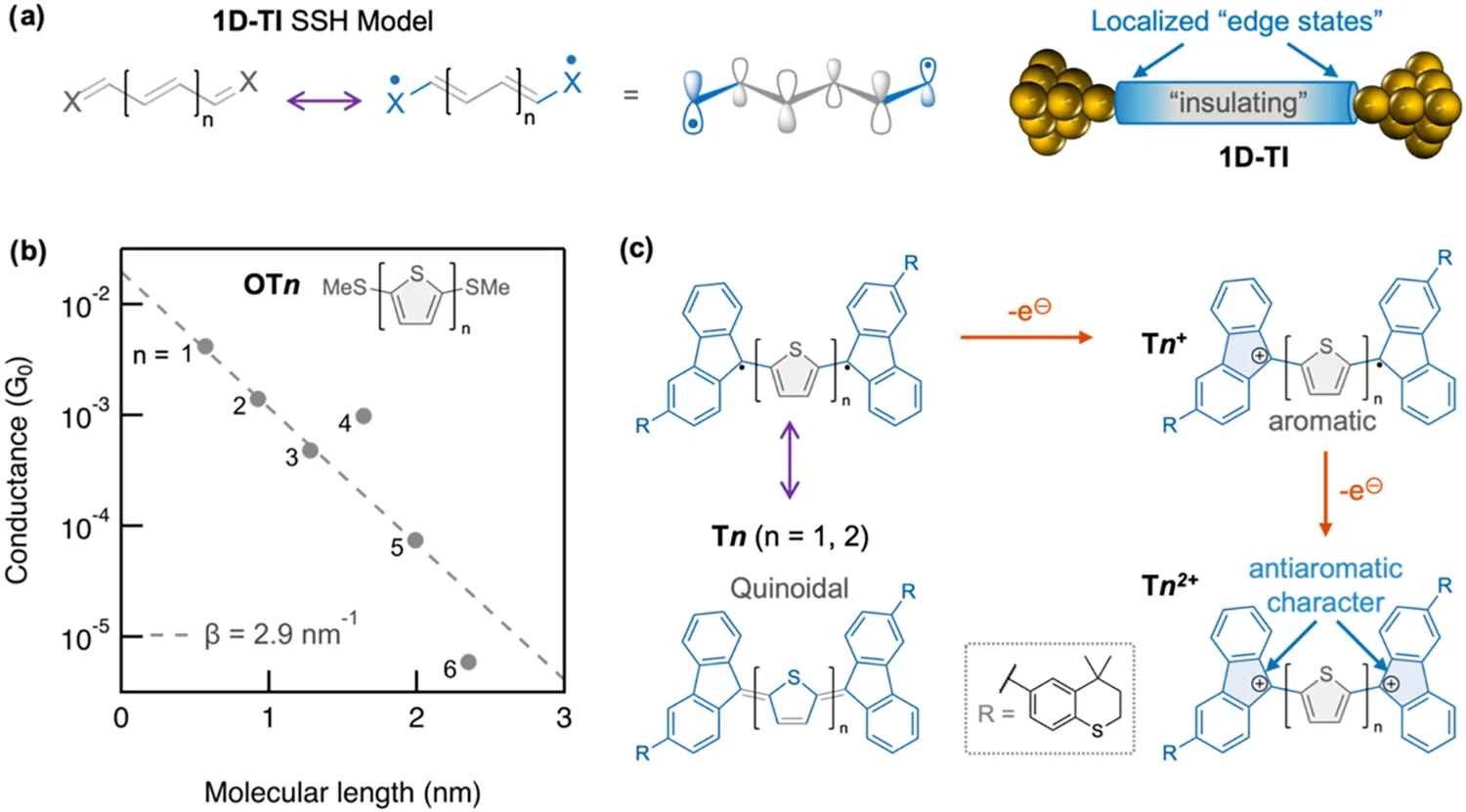
(a) Illustration showing edge states created in molecular wires (X = carbon or nitrogen). (b) Previous studies: Plot of conductance as a function of length of oligothiophene, **OT** ***n***, series where *n* = 1–6.^33^ The conductance values are fit with an exponential (*G* ∼ *e* ^β*L*^) to determine the decay constant (β). (c) Backbone structures of **T** ***n*** molecular wires (*n =* 1, 2 in this study), exhibiting a 1D topological insulator character, as well as antiaromaticity upon oxidation.

In parallel, studies have also revealed that reduced aromaticity enhances transport properties, yet little is known about aromaticity effects of conjugated 1D-TIs, especially multistate conduction systems with *in situ* redox reversibility to induce antiaromatic character.^27–31^ While previous designs of 1D-TIs have focused on diradicaloid character, we recognize that the aromaticity of molecular wires also affects the alignment of transport orbitals relative to the electrode Fermi level (*E*_F_). Thus, it is possible that tuning antiaromaticity could enable more efficient electron transport within a single-molecule junction (aromaticity shifts transport orbitals away from resonance, and greater antiaromatic character draws them closer).^28^ Indeed, conductance changes between aromatic and antiaromatic states have been quantified within an individual redox-active molecule in a junction, but 1D-TI behavior was not observed. The *in situ* electrochemical methods only achieved switchable transport behavior through two-state reversible aromatic character transitions.^29^ Moreover, molecular 1D-TIs based on positively charged carbon are less known, even though they have been postulated to exhibit soliton behavior in polyacetylenes.^32^ For these reasons, a robust framework of structure–property relationships to achieve multistate redox transport behavior in single-molecule junctions of 1D-TIs would provide a stepping stone toward controlling switching with external stimuli.

We posit that 1D-TIs can be engineered as stimuli-responsive molecules capable of achieving redox-controlled, multistate transport, representing an unexplored approach to induce switching in single-molecule devices. Here, we focus on quinoidal thiophene-based architectures, leveraging their redox characteristics to engender 1D-TI properties driven by aromaticity-antiaromaticity switching. At the molecular level, it is well understood that architecture, functionality, and connectivity affect transport properties.^33–36^ For example, a fundamental characteristic of oligothiophenes (**OT** ***n***, Figure 1b) is that they exhibit the expected conductance decay behavior as a function of oligomer length (β = 2.9 nm^–1^).^33^ However, changing the transport path by connecting the thiophene moieties to obtain quinoidal architectures—rendering non-aromaticity of the thiophenes—could give rise to vastly different conductance patterns (Figure 1c). Upon undergoing one/two-electron oxidation, the non-aromatic quinoidal systems **T** ***n*** reveal an aromatic thiophene path that serves as the “insulating” component of a 1D-TI. Since antiaromaticity can increase conductance, it is envisioned that the “edge states” can be accessed by the oxidized fluorene flanking units. Such a design could function as a three-state, redox-controlled 1D-TI (*e.g.*, neutral, +1, and +2).

## Results and Discussion

To access the desired quinoidal conjugated path across thiophene and 2,2’-bithiophene backbones, fluorenes were linked through the 9-position as shown in Figure 1b (**T** ***n***). Compounds **T1** and **T2** were synthesized and characterized by conventional methods (see the Supporting Information, SI, for details), and attempts to synthesize **T3** were unsuccessful due to its insolubility. The oxidation potentials of **T** ***n*** were obtained by cyclic voltammetry (CV, Figure 2a, SI Figures S1 and S2). Two distinct oxidation peaks are observed from **T1**, while **T2** exhibits broad, overlapping peaks. Electron paramagnetic resonance spectroscopy shows a peak of greater intensity in **T2** than in **T1** (SI Figure S3), consistent with the slightly enhanced radical character expected from the aromatized thiophene resonance structure (Figure 1c).^19,37^

**Figure 2:**
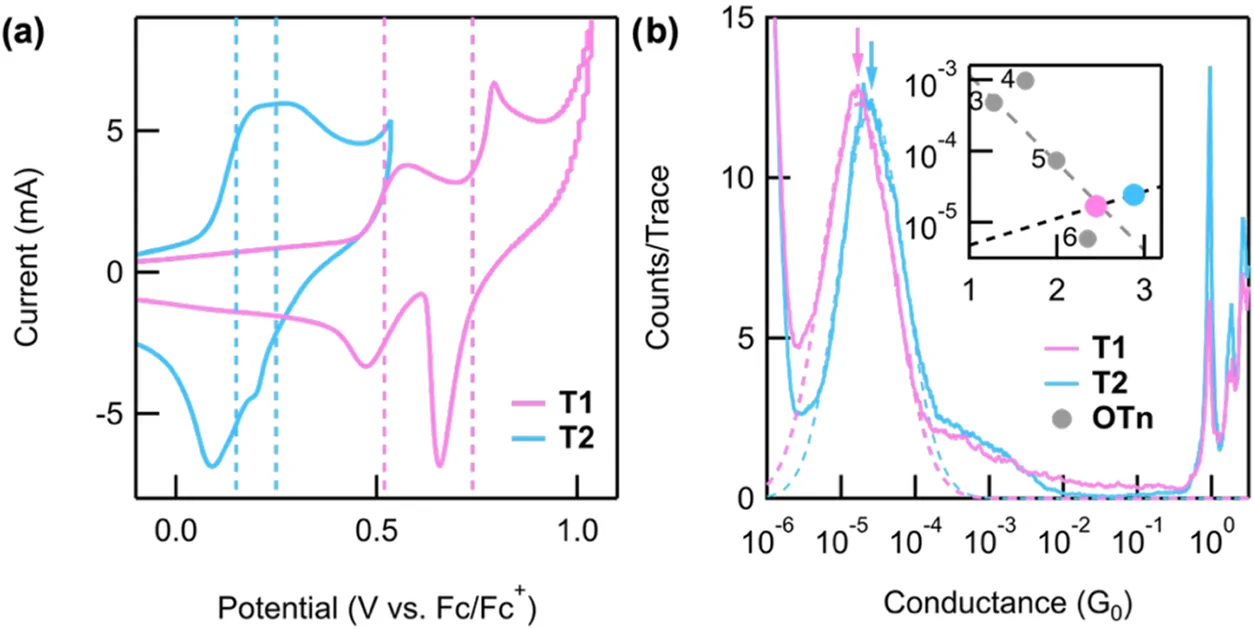
(a) Cyclic voltammetry (CV) measurements show the oxidation waves of **T1** and **T2**, with redox potentials (*E*_1/2_ of 0.52 and 0.74 V for **T1**; and 0.15 and 0.25 V for **T2** vs Fc/Fc^+^). (b) One-dimensional (1D) conductance histograms of **T1** (pink, 2000 traces) and **T2** (blue, 2000 traces) measured at 200 mV in propylene carbonate (PC), along with Gaussian fits to the peaks. Inset: Plot of conductance versus molecular length of **T1** and **T2** (pink and blue points, respectively), determined from Gaussian fits, and oligothiophenes **OT3**–**OT6**. The gray dashed line shows the conductance decay of the **OT** ***n*** series from Figure 1b, and the black dashed line is a fit to the conductance values of **T1** and **T2**.

The molecular conductance of the **T** ***n*** wires was obtained using a customized STM-BJ technique detailed in previous work.^38^ Single-molecule junctions are formed through the repeated formation and breaking of Au point contacts in molecular solutions. The STM tip is first brought into contact with the substrate and then retracted to form a single Au–Au atom contact characterized by a conductance of *G*_0_ = 2e^2^/*h*. Once the contact is broken, a molecule can bind in the resulting gap to form a single-molecule junction. When a bias voltage is applied between the tip and substrate, the resulting current is measured as a function of relative tip–substrate displacement. This procedure is repeated thousands of times, collecting data to provide statistically significant insights into the conductance (current/voltage), reported as 1D and 2D histograms generated without data selection. When using polar solvents in this study (*e.g.*, propylene carbonate, PC), the capacitive and Faradaic background currents are minimized by coating STM tips with Apiezon wax, reducing the exposed surface area to under ∼10 μm^2^,^39^ Pristine tips are used with less polar solvents, such as 1,2,4-trichlorobenzene (TCB) and dichloromethane (DCM).

We measured the transport properties of neutral **T** ***n*** wires using PC as a solvent and applying a tip bias voltage of 200 mV (with the substrate grounded), and the plots are shown in Figure 2b. The **T** ***n*** wires show a reversed conductance decay with increasing molecular junction length, which is attributed to the diradical nature of the molecular systems^17,23,25^ supporting the possibility that these molecules function as 1D topological insulators in their neutral ground state (β = −0.67 nm^–1^, see the inset in Figure 2b). We recognize that the β value is obtained from two points; we hypothesize that the conductance of **T2** (2.6 × 10^–5^ *G*_0_) is higher than that of **T1** (1.7 × 10^–5^ *G*_0_) due to a more pronounced diradical character (SI Figure S3). It is also worth noting that the conductance in the neutral state of the **T** ***n*** wires is only slightly higher than that of oligothiophene wires of similar length (inset in Figure 2b).^33^ We attribute the low conductance to the slight diradical character that reveals aromatic, low-conductance thiophene units as shown in Figure 2b. To test how the antiaromaticity of the fluorene units (edge states) impacts the transport properties of the **T** ***n*** molecular wires, we assessed three methods to robustly characterize the oxidized species (**T** ***n*** ^**+**^ and **T** ***n*** ^**2+**^): (*i*) photoredox chemistry (**T** ***n*** ^**+**^ only, reversibly), (*ii*) chemical oxidation (**T** ***n*** ^**+**^ and **T** ***n*** ^**2+**^ irreversibly), and (*iii*) electrochemical oxidation (**T** ***n*** ^**+**^ and **T** ***n*** ^**2+**^ reversibly).^28^

The singly-oxidized state was obtained by irradiating **T2** with a 660 nm laser (defocused with intensity of 100 mW cm^–2^), which undergoes electron transfer with an oxidizing agent—bis­(4-*tert*-butylphenyl)­iodonium hexafluorophosphate (**[R**_**2**_ **I]** ^**+**^[PF_6_]^−^)—as shown in Figure 3a. Note that the laser intensity on the tip/substrate region is not sufficiently high to heat the junction. For this experiment, we prepared a solution of **T2** and **[R**_**2**_ **I]** ^**+**^ in a 1:9 ratio and with **T2** at a 0.1 mM concentration in a 3:1 mixture of TCB and DCM following the procedure detailed in a previous work.^40^ The resulting STM-BJ measurements under irradiation at a tip bias of 200 mV yielded the conductance plot in Figure 3b, exhibiting an average conductance of ∼1 × 10^–3^ *G*_0_, which is a factor of 40 higher than that of the neutral species. We attribute the high conductance peak to the monocation **T2** ^+^ and not the twice-oxidized species since the oxidation potential of **[R**_**2**_ **I]** ^**+**^ is not high enough to access such a state. We also note that the conductance reverts to the lower value once the laser is turned off. The formation of **T2** ^+^ is further supported with control experiments—irradiating a solution mixture of **T2** and **[R**_**2**_ **I]** ^**+**^ in a UV-Vis spectrometer shows a significant change in the absorption spectrum (see Figure 3c); and excluding **[R**_**2**_ **I]** ^**+**^ under irradiation does not lead to noticeable changes in the conductance trace (Figure S4). Similar experiments with **T1** were not carried out since its oxidation potential is higher than that of **T2** and cannot be oxidized with **[R**_**2**_ **I]** ^**+**^.

**Figure 3:**
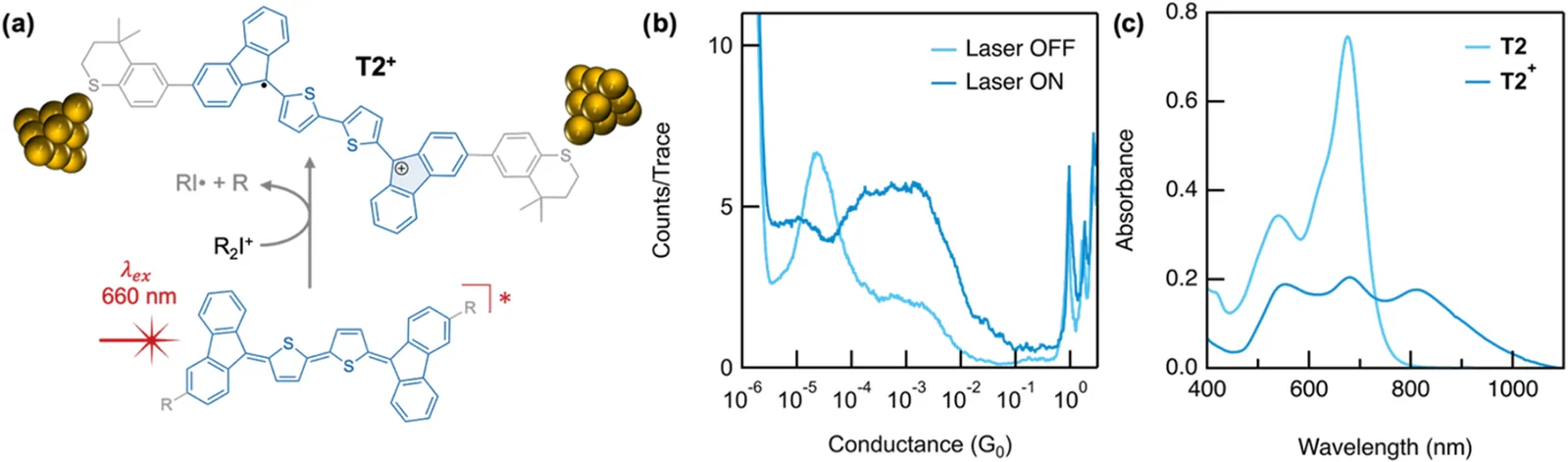
(a) Photoredox reaction during STM-BJ measurements, where **T2** is oxidized upon 660 nm laser irradiation. *R*_2_I^+^ is an iodonium salt (R: 4-*tert*-butylphenyl; counterion: [PF_6_]^−^) 1:3 mixture of DCM and TCB at a concentration of 100 μM, and a 9:1 ratio of R_2_I^+^:**T2**. (b) 1D conductance histograms of **T2** showing conductance changes with and without 660 nm laser irradiation. Each histogram includes 2000 consecutively collected traces. Two-dimensional (2D) conductance-displacement histograms are shown in Figure S5. (c) UV-Vis absorption data for **T2** and **T2** ^**+**^ were obtained by irradiating (660 nm) a mixture of **T2** and R_2_I^+^.

To access higher oxidation states, we turn to chemical oxidation. The first oxidation state (**T2** ^**+**^) can be obtained using one equivalent of the oxidant [Fc]^+^[PF_6_]^−^ (half-wave potential *E*_1/2_ = 0.17 V *vs* Fc/Fc^+^), while the first oxidation state of **T1** and second oxidation **T2** ^**2+**^ was obtained using one and two equivalents of tris­(4-bromophenyl)­ammoniumyl hexachloroantimonate (BAHA, half-wave potential *E*_1/2_ = 0.74 V *vs* Fc/Fc^+^), respectively. Junctions formed with **T2** ^**+**^ and **T2** ^**2+**^ show two distinct conductance peaks with average values that are higher than the neutral state (Figure 4a). The **T1** ^**+**^ molecule junction exhibits a conductance of 1.2 × 10^–4^ *G*_0_, which is 7 times higher than the conductance of the quinoidal **T1**. Due to mismatched redox potentials, the **T1** ^**2+**^ state was not accessible by *in situ* chemical oxidation with BAHA within the STM-BJ measurements. In the chemically oxidized **T2** ^**+**^ molecule, however, we again observe a broad conductance peak around 1.0 × 10^–3^ *G*_0_, akin to the value obtained by photoredox chemistry, confirming that the molecule was singly oxidized. In turn, **T2** ^**2+**^ shows a markedly high conductance value of 0.01*G*_0_, which is ∼400 times higher than **T2**—a molecule with a length of 2.9 nm. It is important to highlight how antiaromaticity leads to such drastic changes in conductance in these molecules, especially **T2**. The nucleus-independent chemical shift (NICS) values (Figures S8 and S9) and anisotropy of the induced current density (AICD) plots show the aromaticity variations within the various oxidation states, 0, +1, +2 (Figures S10 and S11). The central quinoidal thiophene rings exhibit non-aromatic character in the neutral state, as expected, and then become aromatic in both mono- and dicationic states. These observations align with the structural evolution of the 1D-TI presented in Figure 1c as a function of oxidation state: the quinoidal backbone becomes aromatic (low conductance/insulating), and the terminal five-membered rings become 4π-electron antiaromatic units (edge states).

**Figure 4:**
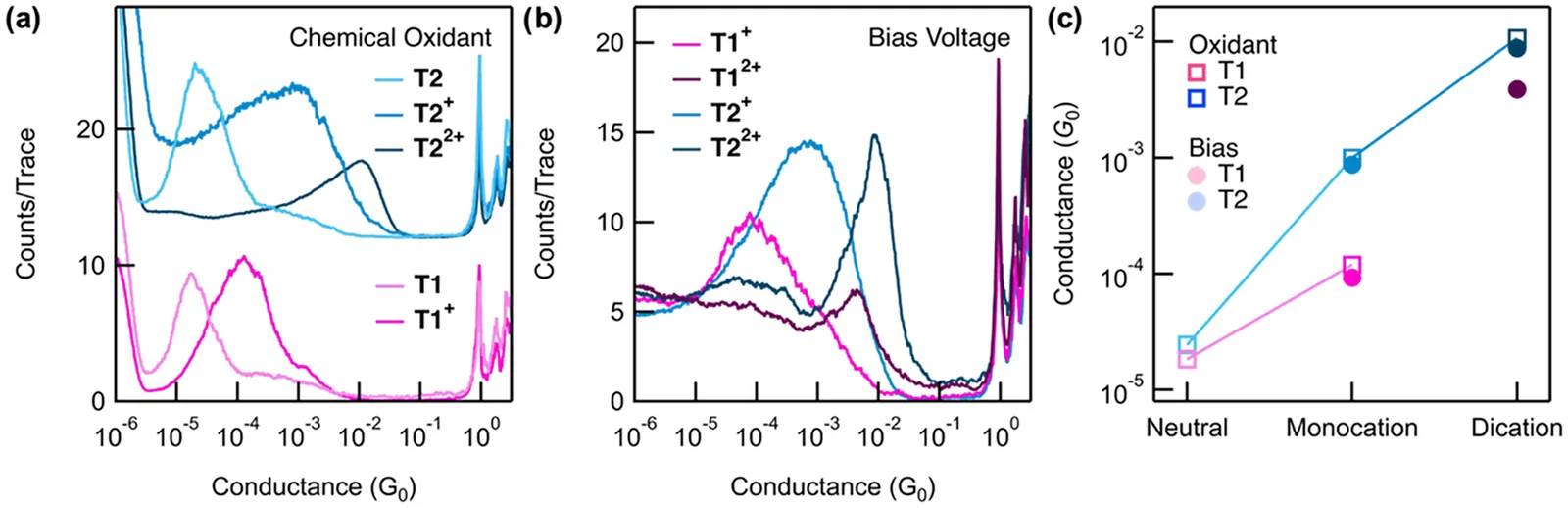
(a) 1D conductance histograms of **T1** and **T2** and the chemically oxidized units using BAHA and [Fc]^+^[PF_6_]^−^. The measurements were conducted at an applied bias voltage of 200 mV. 2D conductance-displacement histograms are shown in Figure S6. All histograms include 2000 traces, excluding the one with **T2** ^**+**^, which is generated from 500 traces as the molecule reverts back to the neutral state during the measurement. (b) 1D conductance histograms of the electrochemically oxidized **T1** and **T2** obtained by applying tip bias voltage during the STM-BJ measurements. Tip bias voltages of 900 mV and 1,300 mV were applied to obtain the **T1** ^**+**^ and **T1** ^**2+**^ states, respectively, and 500 mV and 1,200 mV for the **T2** ^**+**^ and **T2** ^**2+**^ states, respectively. All histograms include at least 2000 traces. 2D conductance-displacement histograms are shown in Figure S7. (c) Conductance values determined from Gaussian fits to the 1D histograms of conductance peaks from panels (a, b). The fitting errors are within the size of the markers.

Considering that the **T1** ^**2+**^ state was not accessible by either photoredox chemistry or chemical oxidation methods, we turn to *in situ* oxidation through electrochemistry, varying the tip bias voltage in the STM-BJ. A gold tip coated with Apiezon wax is used to generate an asymmetric voltage drop across the tip–substrate junction by creating a significant difference in the exposed surface area between the coated tip and the bare Au substrate.^41^ At a bias voltage of 200 mV, which is below the first oxidation potential of both **T1** and **T2**, we form molecular junctions in the neutral state. The first and second oxidation of **T1** occur at a tip bias of 900 mV and 1300 mV, respectively, while those of **T2** are at 500 mV and 1200 mV (see Figure S4c for additional data). When calibrated with the redox potential of ferrocene *in situ*, these tip biases are consistent with the potentials obtained from *ex situ* CV measurements (Figure S2). The resulting conductance plots now provide a complete picture of all oxidized species (Figure 4b). The conductance values are compiled and plotted in Figure 4c—**T1** ^+^ and **T1** ^**2+**^ exhibit conductance of ∼9 ×10^–5^ *G*_0_ and ∼5 ×10^–3^ *G*_0_, while **T2** ^**+**^ and **T2** ^**2+**^ are ∼9 × 10^–4^ *G*_0_ and ∼9 × 10^–3^ *G*_0_, respectively. Importantly, all methods to access the oxidized species are consistent and, notably, the longer **T2** ^**2+**^ displays the highest conductance. Taken together, our measurements show unequivocally that the conductance increase going from **T1** to **T2** is higher in the oxidized systems than in the neutral state (*e.g.*, conductance trend based on oxidized state: 2+ > 1+ > 0). Comparing the neutral and the oxidized species, the key difference is that the fluorene moiety gains antiaromatic character, with increasing aromaticity of the central thiophene rings, further supporting the dominant influence of antiaromaticity on the 1D-TI model.

We finally rationalize our experimental findings by performing DFT-based transmission calculations for **T1** and **T2** within the NEGF formalism using the FHI-aims and the AITRANSS package in the neutral and dication states.^42–46^ First, we optimized the geometry of each molecule with two Au atoms bound to the thiochroman linkers using the PBE functional,^47^ and then attached Au_59_ clusters to represent the electrodes, as shown in Figure 5a,b. We use the AITRANSS package to compute the transmission of these junctions first in the neutral state, as shown in Figure 5c. We see that the transmission of **T2** at *E*_F_ is higher than that of **T1**, consistent with the higher conductance observed experimentally. We next calculate the transmission of **T1** ^**2+**^ and **T2** ^**2+**^ using arrays of point charges to maintain a net 2+ charge on the molecule when attached to the electrodes, as we have done previously.^17^ We find that the transmission for both molecules shows a resonance very close to the *E*_F_, and the conductance of both molecules increases substantially when compared to the neutral case. Given errors inherent to DFT when using low-rung functionals,^42,48^ and the proximity of the resonance to the Fermi energy, quantitative comparisons between experiment and theory are incongruent; however, our calculations clearly show the reversed conductance trend going from **T1** to **T2** in the neutral state and a drastically large increase in conductance upon oxidation.

**Figure 5:**
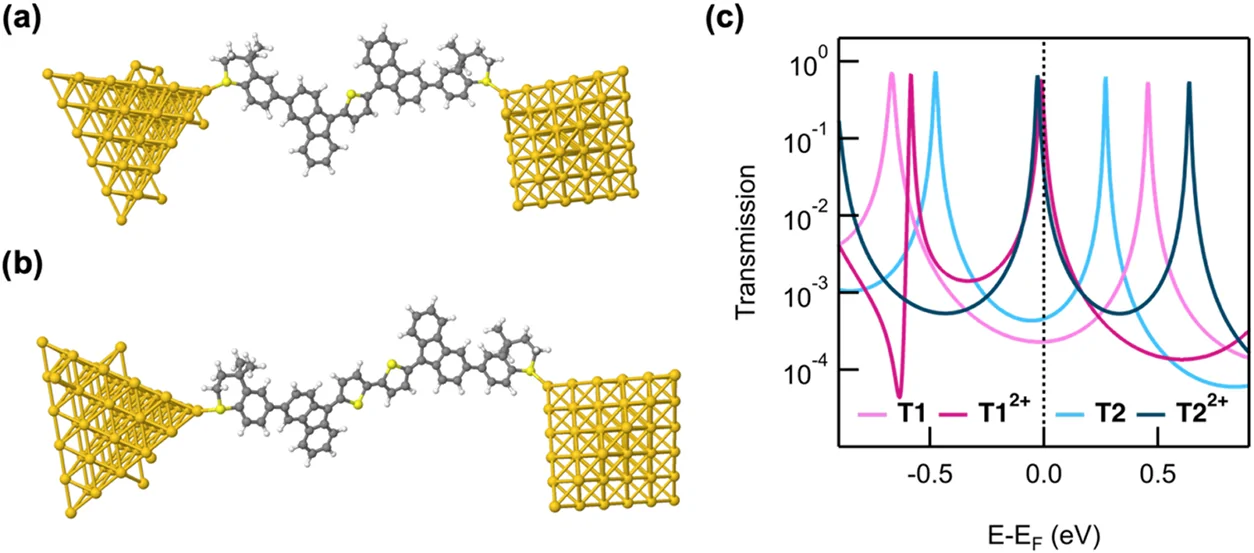
DFT optimized single-molecule junction geometries of (a) **T1** and (b) **T2**. (c) DFT-based transmission function calculated for **T1** and **T2** in the neutral and doubly oxidized states.

## Conclusion

We designed and investigated the conductance behavior of single-molecule 1D-TI wires (**T** ***n***), with tunable multistate conductance controlled electrochemically to test the impact of antiaromaticity. Our STM-BJ measurements show that neutral **T2** is more conducting than T1, consistent with the behavior of 1D-TIs. To further enhance their conductance, we explored the effect of (anti)­aromatic character modulation on electron transport, as evidenced by DFT calculations (including NICS and AICD). Through optical, chemical, and electrochemical oxidation, our observations show that the **T** ***n*** systems display the classical behavior of Su-Schrieffer-Heeger topological insulators. Notably, the doubly oxidized state **T2** ^2+^ exhibits a conductance value that is 400 times higher than that of its neutral counterpart. These findings establish a new approach to enhancing molecular conductance through an antiaromatic character, providing a strategy for designing high-performance molecular conductors.

## Supplementary Material



## Notes

Chemical structures, STM-BJ measurements method, electrochemical characterization, additional experimental data, DFT calculations, synthetic details, and NMR spectra (PDF)
