# Supplementary material for: Redox-Driven Antiaromaticity Enables Multistate Conductance in 1D Topological Insulators
Source: J Am Chem Soc. 2026 Sep 7;148(37):40536–42. doi: 10.1021/jacs.6c15721 (PMC13614195; doi:10.1021/jacs.6c15721)
Supplement: Supplementary file 1 [file ja6c15721_si_001.pdf]

SUPPLEMENTARY INFORMATION

for

Redox-Driven Antiaromaticity Enables Multi-State Conductance in 1D Topological Insulators

Woojung Lee[1], Wanzhuo Shi[1,2], Junho Kwon[1], Jonathan Low[1], Sujun Wei[3], Xiaodong Yin[1], Luis M. Campos[1]*, Latha Venkataraman[1,3,4]*

[1]*Department of Chemistry, Columbia University, New York, New York, 10027, United States*

[2]*Institute of Science and Technology Austria, Am Campus 1, Klosterneuburg, 3400 Austria*

[3]*Department of Chemistry, Queensborough Community College of the City University of New York, Bayside, New York 11364*

[4]*Department of Applied Physics and Applied Mathematics, Columbia University, New York, New York 10027, United States*

Email: latha.venkataraman@ist.ac.at; lcampos@columbia.edu

Contents:

1. **STM-BJ Measurements Method**
2. **Electrochemical Characterization**
3. **Additional Experimental Data**
4. **DFT Calculations**
5. **Synthetic Details and NMR spectra**

1. STM-BJ measurements method

The conductance of **T*n*** wire junctions was studied using the scanning tunneling microscope-based break junction (STM-BJ) technique.[1-3] A piezo actuator precisely controls the movement of the Au tip relative to an Au substrate to form and break Au point contacts. The voltage (V) and current (I) across the junction were continuously monitored while pulling the tip away from the substrate at 20 nm/s at a sampling rate of 40 kHz, enabling the calculation of conductance (G = I/V). To minimize capacitive and Faradaic background currents when using the polar solvent propylene carbonate (PC), the Au tip was coated with Apiezon wax.[4] The significant difference in exposed surface areas between the coated tip and the bare Au substrate creates an asymmetric voltage drop across the tip-substrate junction.[5] This two-electrode system of our STM-BJ setup has been thoroughly validated for its precise control of electrochemical potential, as illustrated in **Figure S1**.[6-10] In our *in situ* cyclic voltammetry (CV) measurements, the Au tip is positioned within 1 μm of the substrate. A dense double layer forms on the tip, enabling charge transfer from the molecule to the tip. These measurements are effectively micro-electrode CVs, characterized by a sigmoidal curve rather than the typical "duck-shaped" curves observed in *ex situ* CV measurements. To ensure the reliability of our results, we compared the *in situ* CV of ferrocene with its *ex situ* CV (**Figure S1**).

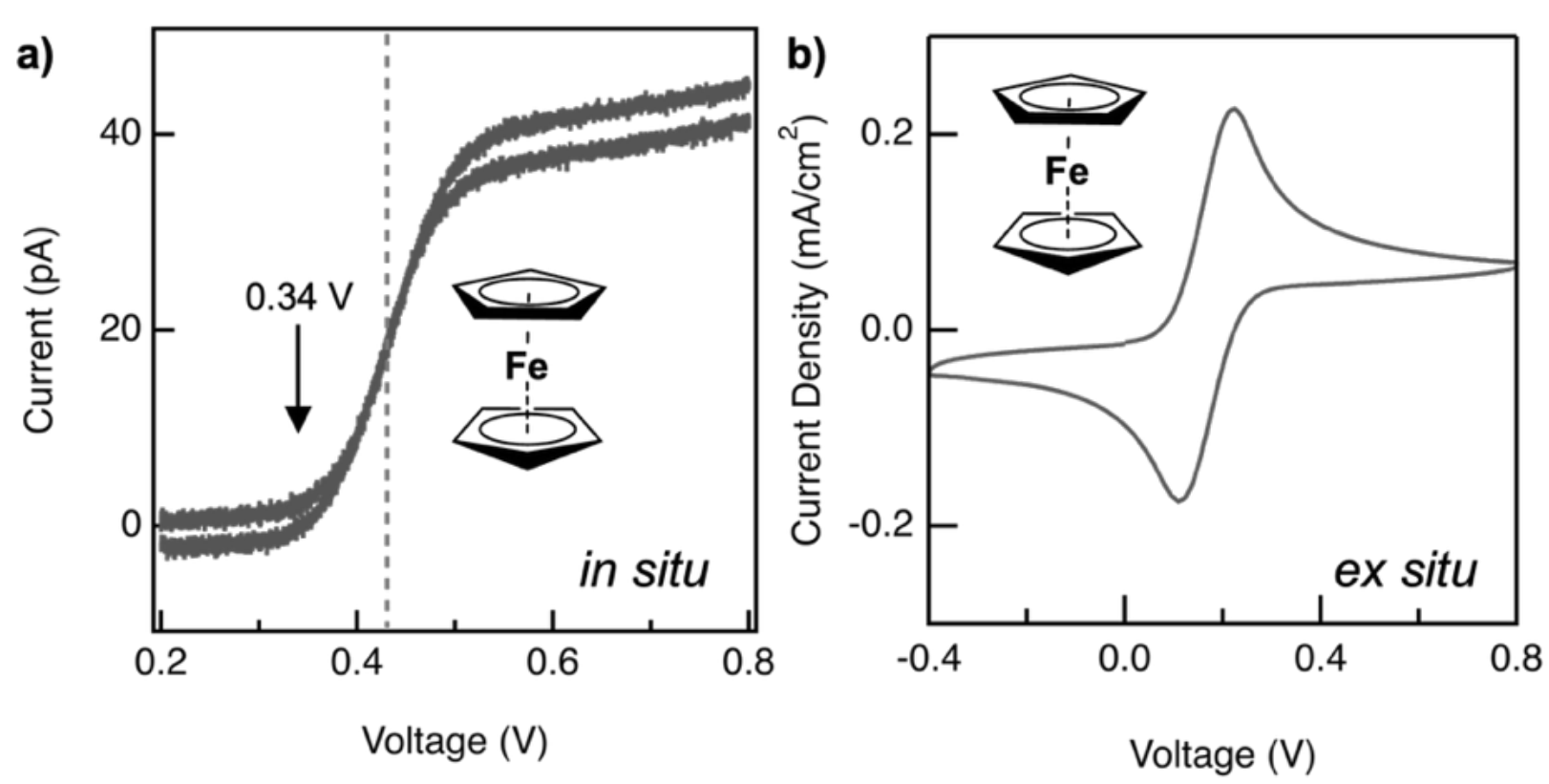

Figure S1. (a) *In situ* cyclic voltammetry (CV) was performed on a 50 μM ferrocene solution in propylene carbonate (PC) using a two-electrode STM-BJ setup. Due to the absence of an electrolyte and the use of a microelectrode, the characteristic "duck" shaped peaks were not observed, as the oxidation process was not diffusion-limited. (b) Ex *situ* CV measurements were conducted on a 1 mM ferrocene solution in PC with 0.1 M $TBAPF_6$, employing a standard DC potentiostat. The recorded potentials were referenced against an $Ag/AgNO_3$ electrode, allowing calibration of a tip potential of 0.34 V in the two-electrode system to approximately 0 V vs. $Ag/AgNO_3$.

2. Electrochemical Characterization

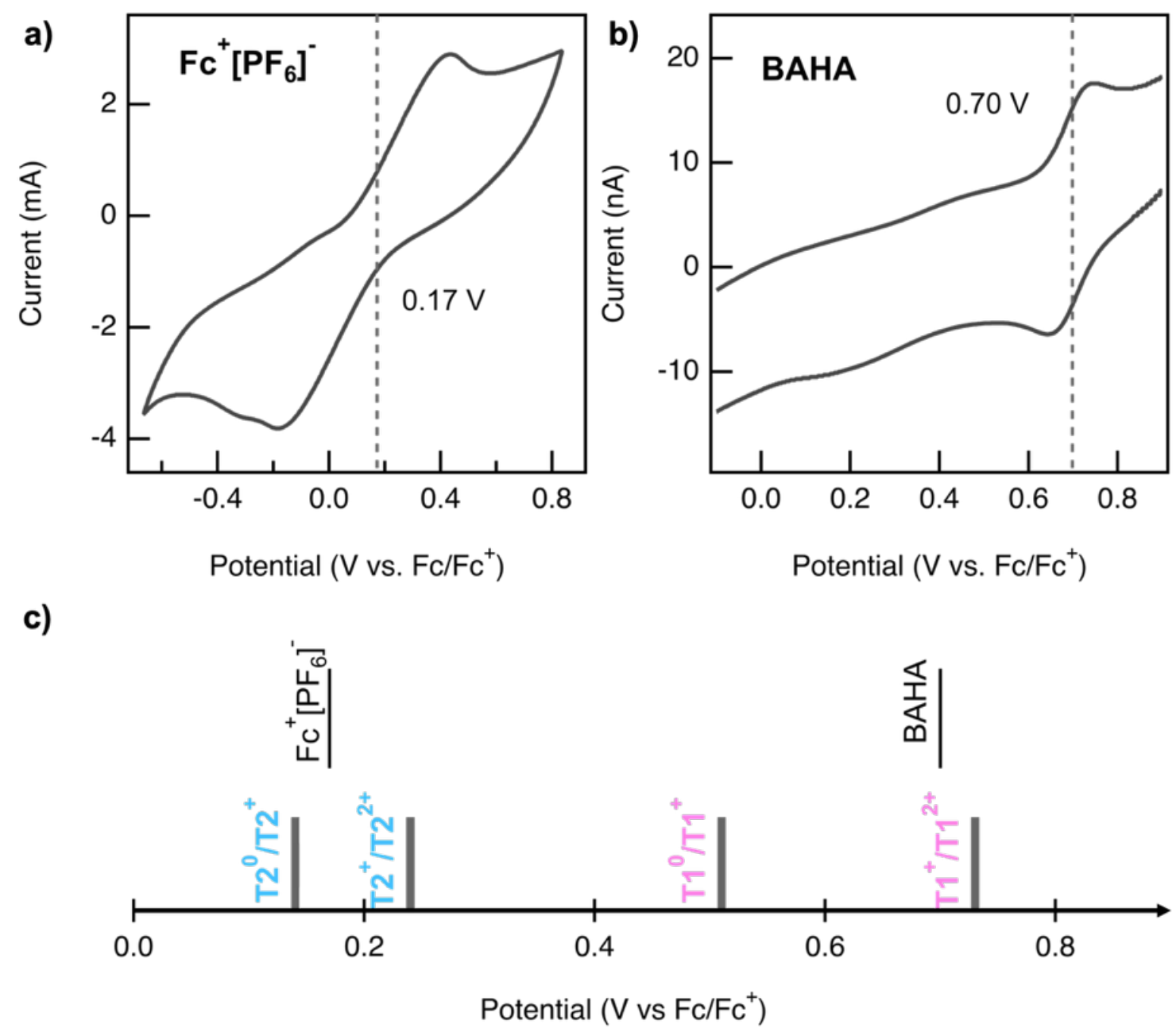

Figure S2. CV measurements of **a)** $Fc^{+}[PF_6]^{-}$ and **b)** BAHA. **c)** Relative redox determined from *ex situ* cyclic voltammetry (CV) measurements. Measurements were performed on a Biologic SP-240 potentiostat using an $Ag/AgNO_3$ reference electrode, Pt wire counter electrode and glassy carbon working electrode. The supporting electrolyte employed was 0.1 M tetrabutylammonium hexafluorophosphate ($TBAPF_6$) in DCM.

3. Additional Experimental Data

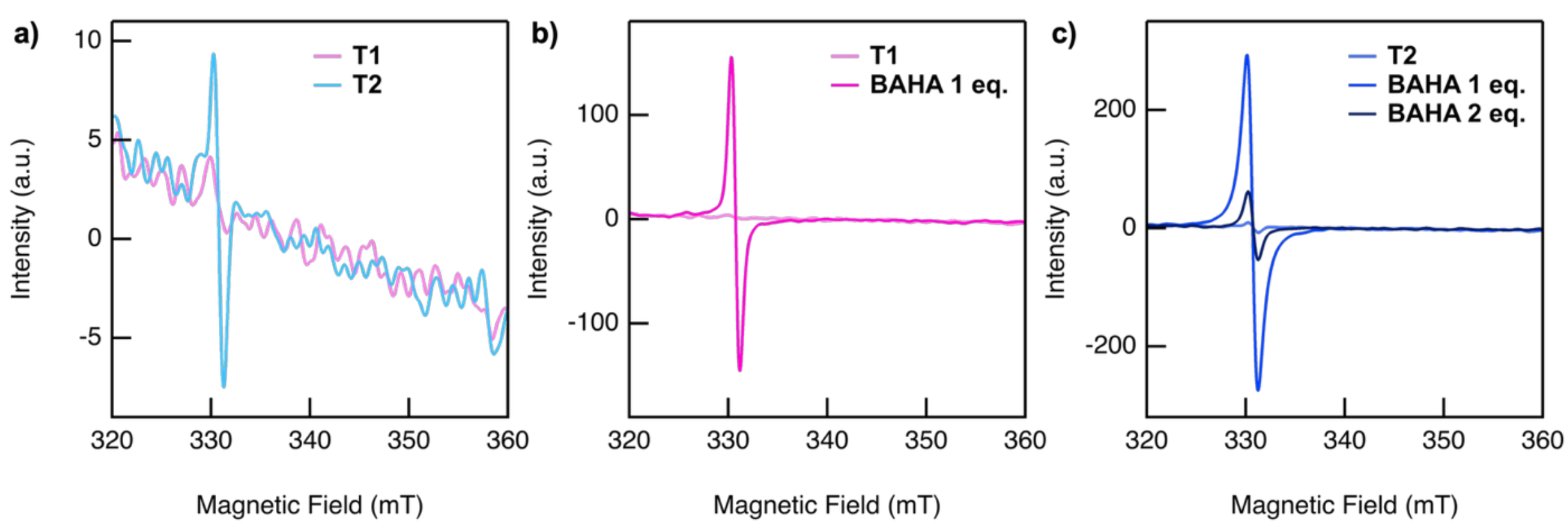

Figure S3. **a)** Electron paramagnetic resonance (EPR) signals of **T1** and **T2** in DCM. **b)** EPR signals of **T1** (500 µM) in DCM with added BAHA. **c)** EPR measurements of **T2** (100 µM) with one and two equivalents BAHA in DCM. All X-band EPR measurements were obtained on an ESR-500 spectrometer at a microwave frequency of 9.437 GHz at a microwave power of 5 mW, a modulation amplitude of 0.2 mT, at a 100 kHz frequency.

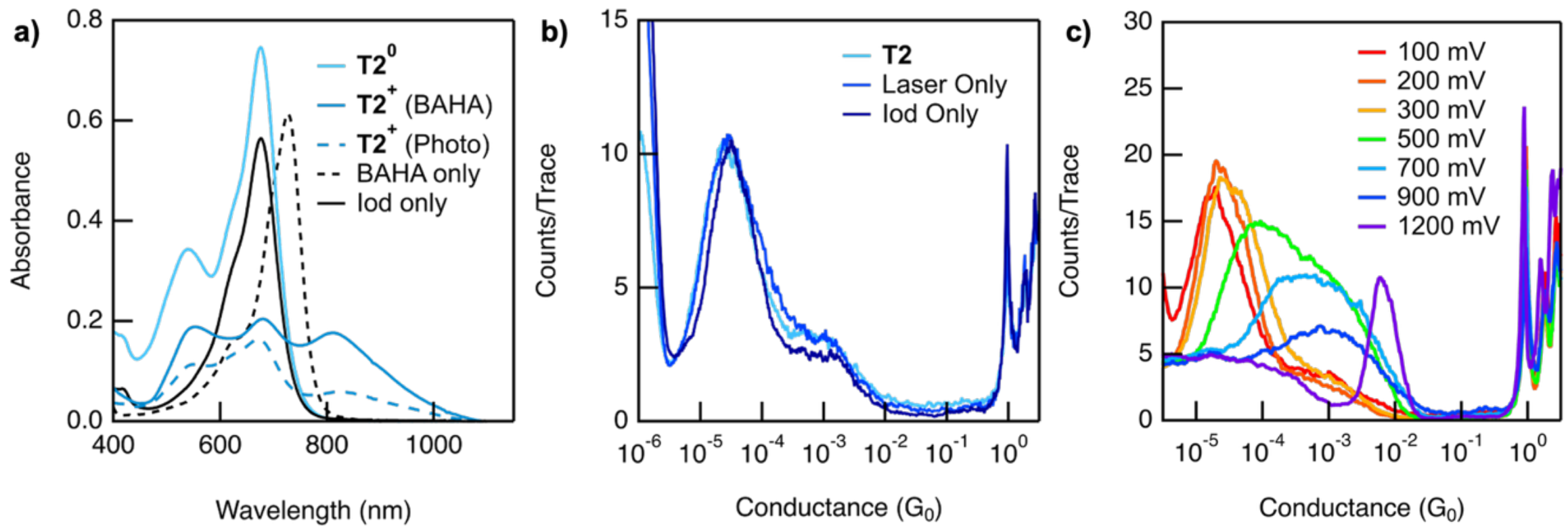

Figure S4. a) UV-Vis absorbance spectra of a 10 μM T2 solution in DCM show no significant changes upon the addition of Iod. However, after laser irradiation at an intensity of 100 mW cm^{-2}, the spectrum indicates a transition from T2^{0} to T2^{+}. When BAHA was added at an equimolar ratio to the T2 solution, the resulting absorbance spectrum closely matched the photooxidation-induced spectrum. Notably, only the solution with added BAHA showed a distinct shift from the original T2 absorbance peak. b) 1D conductance histograms of T2 measured at 200 mV show no peak shift upon adding Iod or irradiating with a 660 nm laser at an intensity of 100 mW cm^{-2}, indicating that neither condition separately oxidizes T2.c) Bias dependent data for measurements of T2 in PC with a coated STM Tip. As the bias is increased, we observe the peak of **T2**$^{+}$ and **T2**$^{2+}$.

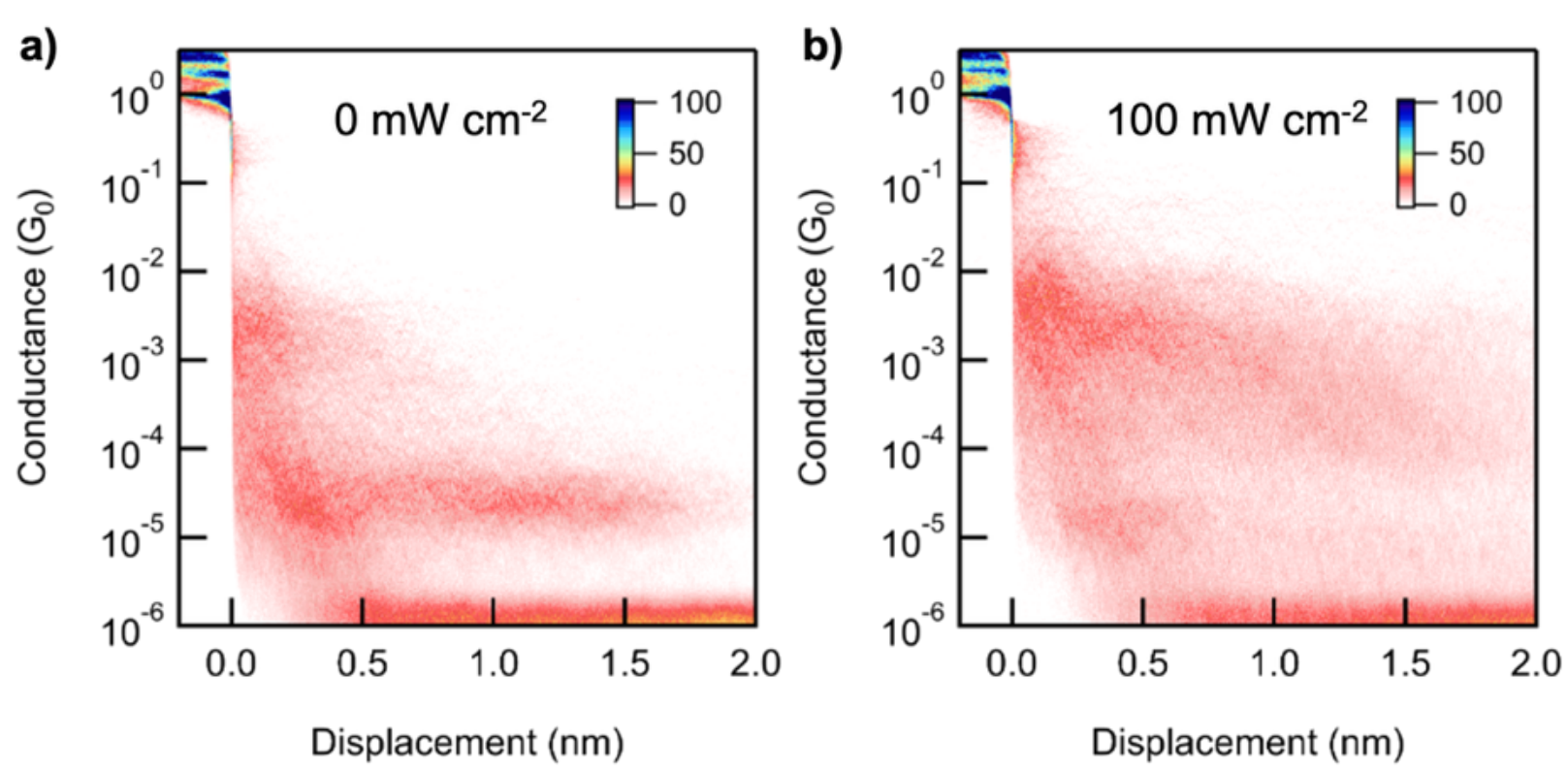

Figure S5. 2D conductance-displacement histograms of **T2** under a) no light and b) irradiation with a 660 nm laser at 100 mW cm^{-2}.

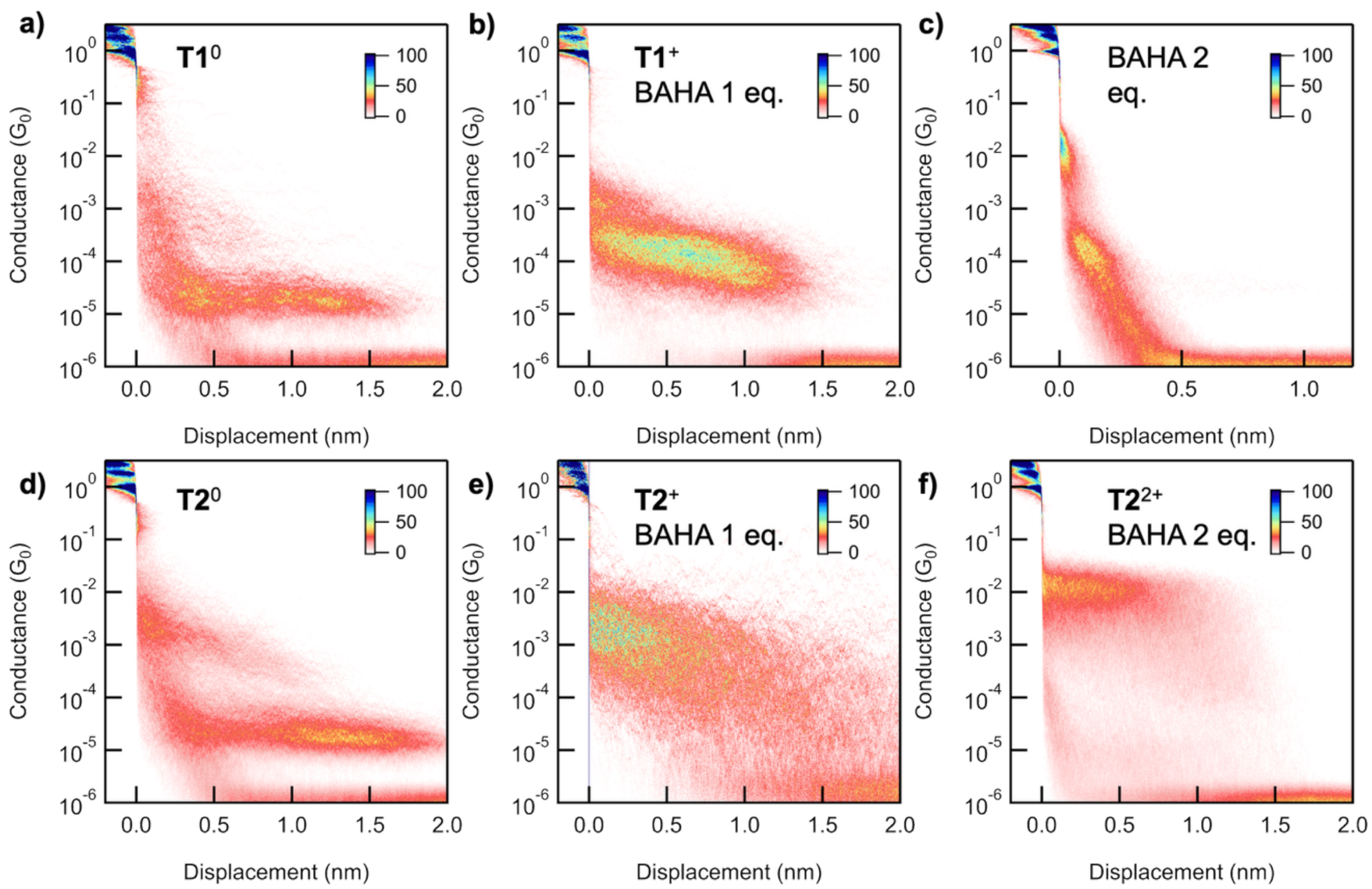

Figure S6. 2D conductance-displacement histograms of **T1** a) to c) and **T2** d) to f) with varying equivalents of added BAHA.

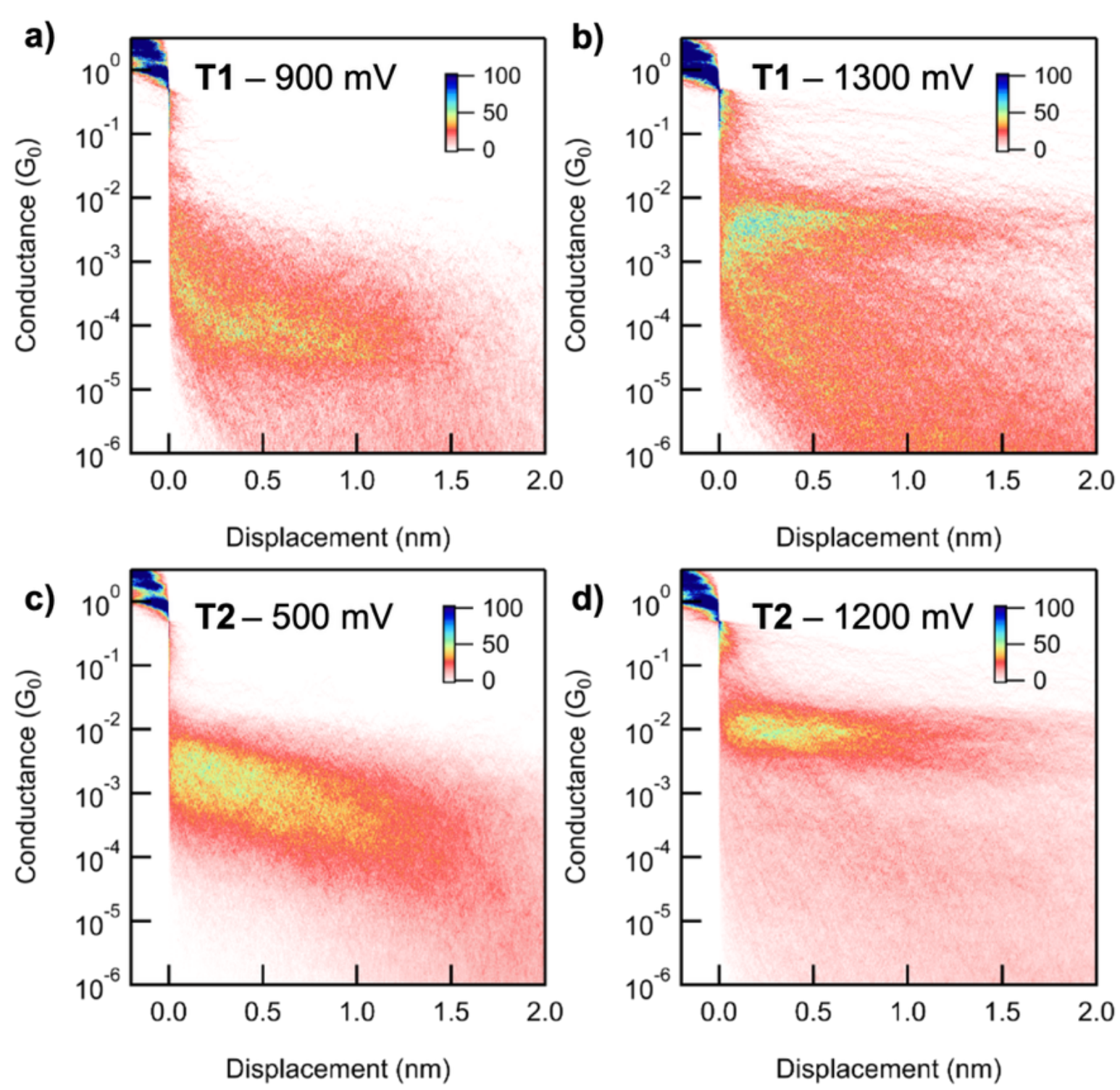

Figure S7. 2D conductance-displacement histograms of **T1** at applied bias voltages as indicated in the figure.

4. DFT calculations

Transmission Calculations: DFT calculations were conducted using both closed-shell and open-shell Kohn-Sham formulations, as implemented in the FHI-aims software.[11,12] A non-empirical generalized gradient approximation (PBE) was used for the exchange-correlation functional,[13] incorporating scalar relativistic corrections to the kinetic energy at the atomic zeroth-order regular approximation level.[14] The Kohn-Sham states were described using an all-electron basis set with tight computational parameters, corresponding approximately to double-zeta plus polarization quality for molecular atoms and double-zeta quality for the Au atom. The energy-dependent transmission functions were calculated using the AITRANSS transport package within the non-equilibrium Green's function (NEGF) formalism.[12] Additionally, the gold electrodes were modeled as tetrahedral clusters of Au atoms with an interatomic distance of 2.88 Å.

NICS Calculations: Changes in aromaticity during oxidation processes were determined using DFT calculations on **T1**, **T2** molecules and their mono-/di-valent oxidized states. The nucleus-independent chemical shift (NICS) values of corresponding molecular systems are presented in Figure S8 for selected rings and also illustrated as 2D contour plots in Figure S9 for more intuitive understanding of aromaticity variations in different rings. Positive NICS values indicate antiaromaticity, and the more positive the value, the stronger the antiaromatic character; conversely, negative NICS values indicate aromaticity, and the more negative the value, the stronger the aromatic character. As shown in the figures, the central quinoidal thiophene ring gradually transforms from non-aromatic character in neutral state to significant aromatic thiophene ring characteristics in mono- and di-cationic states. Meanwhile, the five-membered rings in terminal fluorene units exhibit markedly enhanced anti-aromaticity, accompanied by reduced aromaticity or even emerging anti-aromaticity in adjacent benzene rings. These observations align with the structural evolution during oxidation: the quinoidal structure progressively converts to an aromatic configuration, while the terminal five-membered rings become 4π-electron anti-aromatic systems.

AICD (Anisotropy of the Induced Current Density) of T1, T2 and their oxidized species were also illustrated at B3LYP/6-31G* level of theory. From the figures, it can be observed that in the neutral state of **T1** and **T2** molecules, the benzene rings of the terminal fluorene units exhibit significant aromatic diamagnetic ring currents, while the central quinoidal thiophene ring shows no distinct ring current characteristics. Upon oxidation to mono- and di-cationic states, the most

notable changes are as follows: the π-electrons of the five-membered rings in the terminal fluorene units display pronounced paratropic ring currents, reflecting typical anti-aromaticity, accompanied by emerging paratropic currents in adjacent benzene rings. In contrast, the central thiophene ring develops a diamagnetic ring current, demonstrating aromatic features. These trends align consistently with the calculated NICS results.

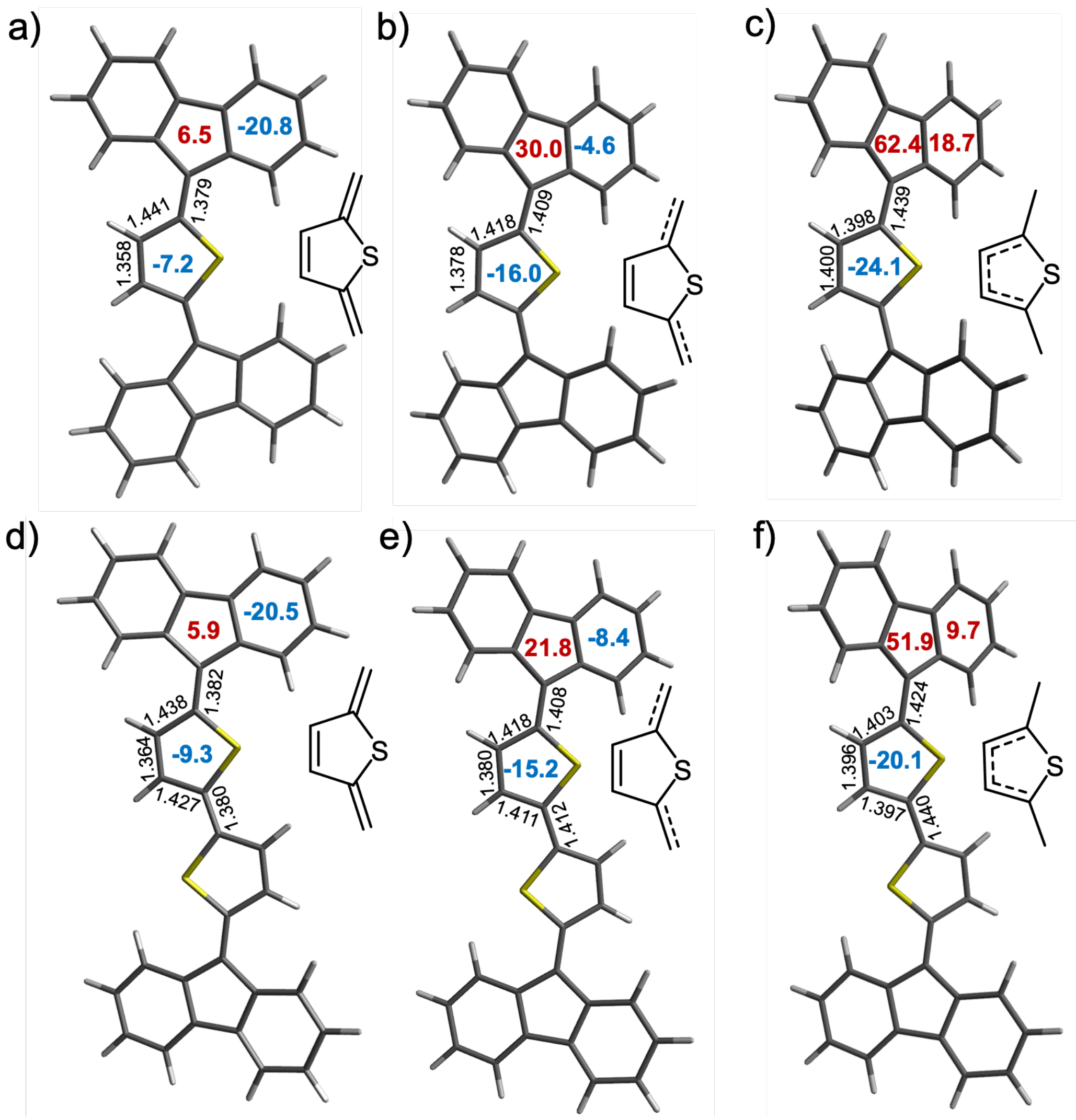

Figure S8. Optimized molecular structures of **T1** in its a) neutral, b) radical cation, and c) dication states, and **T2** in its d) neutral, e) radical cation and f) dication states, along with the calculated NICS(1)zz values of the selected rings and the bond lengths of the key C–C bonds. Upon stepwise oxidation, the central five-membered ring in the two terminal fluorene units exhibits progressively enhanced antiaromaticity, while the central thiophene ring transforms from a quinoid structure in the neutral state into an aromatic ring in the oxidized states.

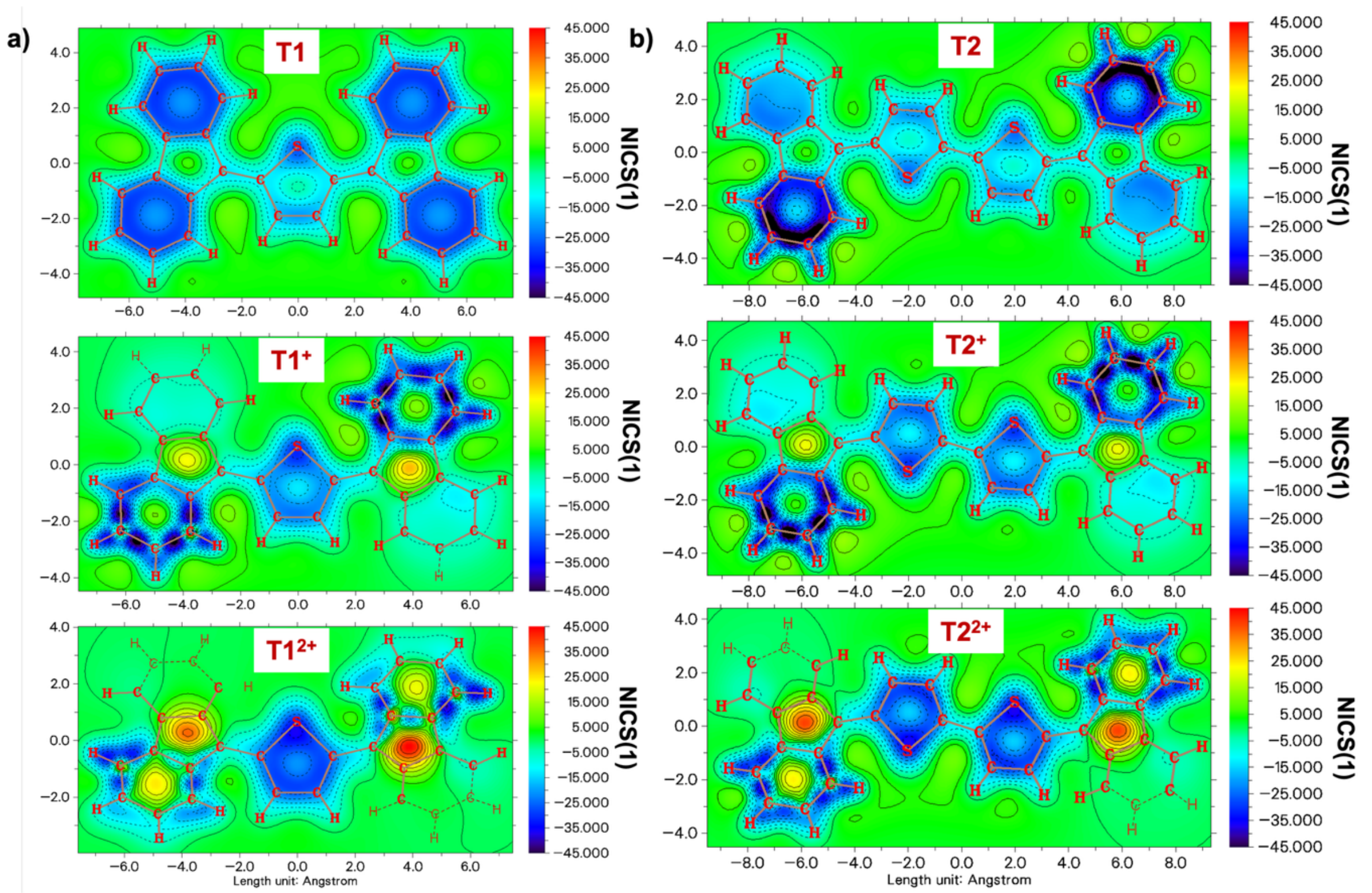

Figure S9. Colored contour map of NICS(1) of a) **T1**, **T1**$^+$ and **T1**$^{2+}$, and **b) T2**, **T2**$^+$, and **T2**$^{2+}$.

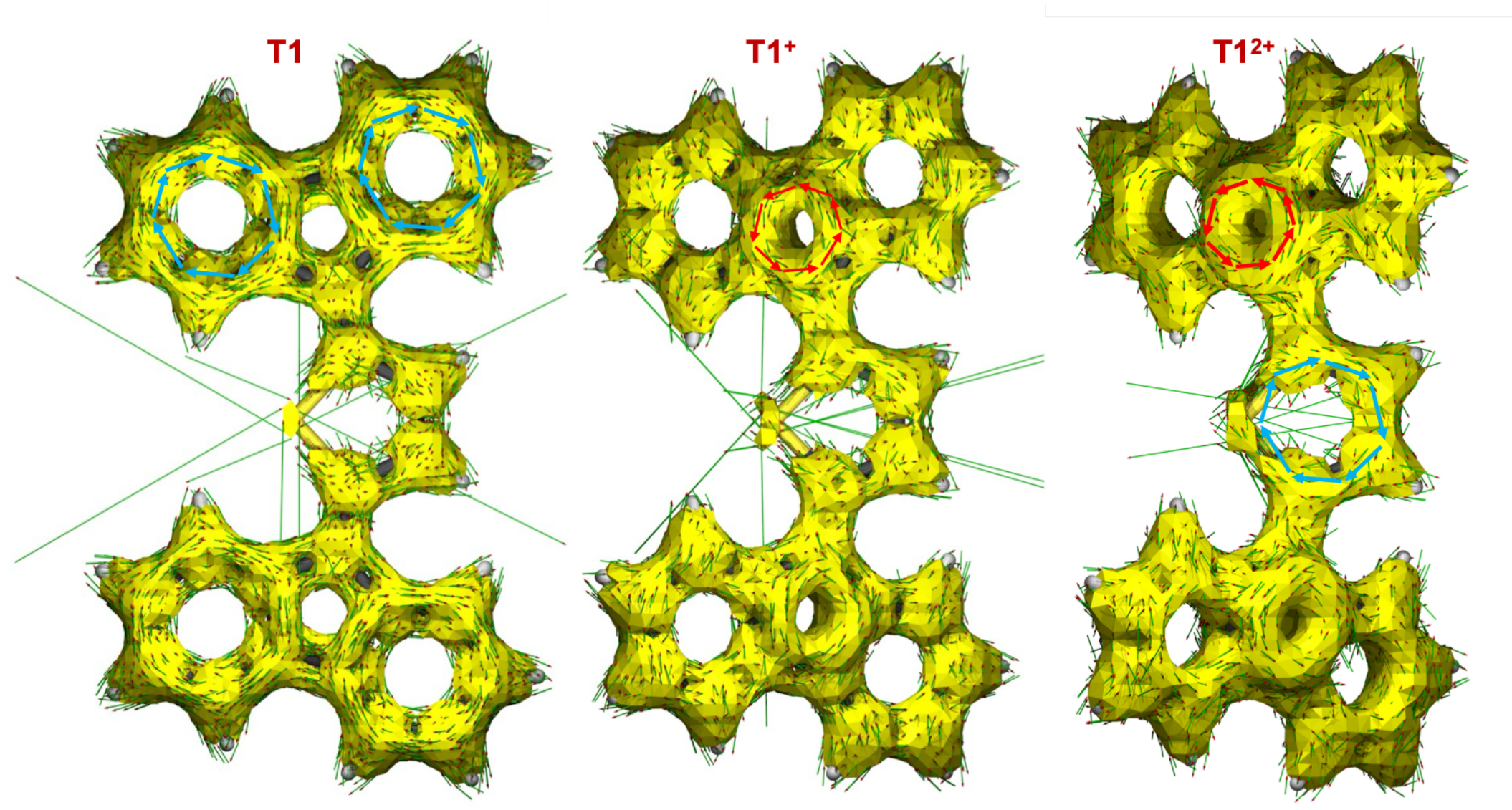

Figure S10. Calculated anisotropy of the induced current density (AICD) plots of **T1** in its neutral, radical cation, and dication states. Clockwise and counterclockwise circulation represent diatropic (aromatic) and paratropic (antiaromatic) ring currents, respectively. In the neutral state, the benzene rings of the two terminal fluorene units exhibit typical diatropic ring currents. Upon oxidation, the paratropic ring current within the five-membered ring of each fluorene unit is enhanced. The central thiophene rings show progressively intensified diatropic ring current with increasing oxidation level.

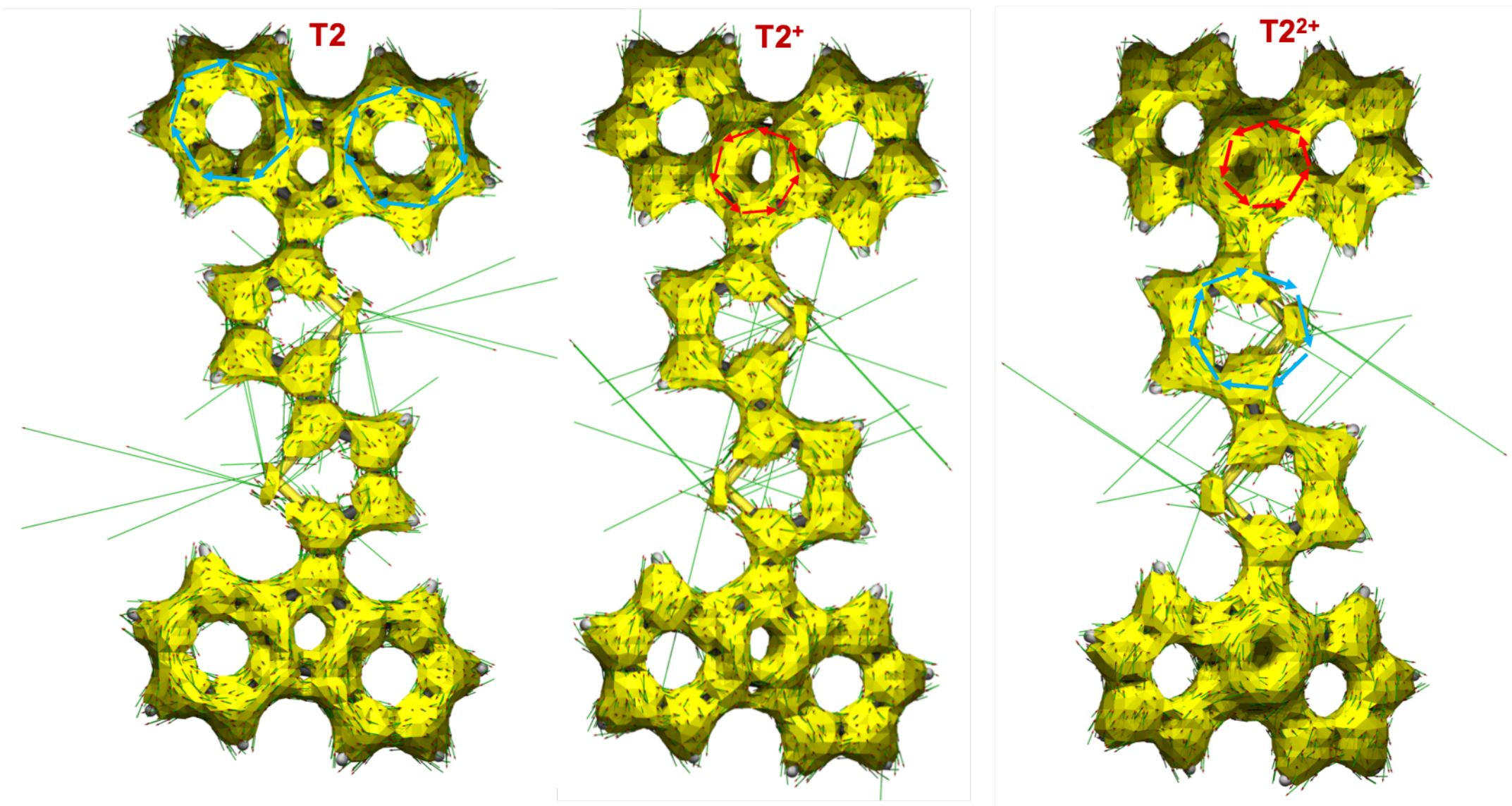

Figure S11. Calculated anisotropy of the induced current density (AICD) plots of **T2** in its neutral, radical cation, and dication states. Clockwise and counterclockwise circulation represent diatropic (aromatic) and paratropic (antiaromatic) ring currents, respectively. In the neutral state, the benzene rings of the two terminal fluorene units exhibit typical diatropic ring currents. Upon oxidation, the paratropic ring current within the five-membered ring of each fluorene unit is enhanced. The central thiophene rings show progressively intensifie d diatropic ring current with increasing oxidation level.

Table S1. Optimized structure of **T1** and **T2** in all oxidation states for NICS calculations.

**T1**							
Element	Coordinates (angstroms)			Element	Coordinates (angstroms)		
	X	Y	Z		X	Y	Z
C	1.3021	-0.5965	0.0000	C	-6.2418	-1.0945	0.0003
C	0.6790	-1.8954	0.0000	C	-6.3108	-2.4884	0.0008
C	-0.6790	-1.8954	0.0000	C	-5.1357	-3.2441	0.0013
C	-1.3021	-0.5965	0.0000	C	-3.8819	-2.6276	0.0011
S	0.0000	0.6204	0.0000	C	-2.6787	2.3509	-0.0007
C	2.6445	-0.2824	-0.0001	C	-3.5133	3.4719	-0.0012
C	-2.6445	-0.2824	0.0001	C	-4.9038	3.3344	-0.0012
C	3.2426	1.0689	0.0003	C	-5.4831	2.0633	-0.0008
C	4.6563	0.9443	0.0004	H	1.2490	-2.8103	0.0000
C	4.9943	-0.4767	-0.0001	H	-1.2490	-2.8103	0.0000
C	3.7894	-1.2301	-0.0004	H	1.6077	2.5041	0.0007
C	-3.2426	1.0689	-0.0003	H	3.0696	4.4629	0.0015
C	-4.6563	0.9443	-0.0004	H	5.5354	4.2178	0.0015
C	-4.9943	-0.4767	0.0001	H	6.5637	1.9528	0.0009
C	-3.7894	-1.2301	0.0004	H	7.1507	-0.4997	0.0000
C	2.6787	2.3509	0.0007	H	7.2762	-2.9854	-0.0010
C	3.5133	3.4719	0.0012	H	5.1916	-4.3286	-0.0018
C	4.9038	3.3344	0.0012	H	3.0081	-3.2643	-0.0015
C	5.4831	2.0633	0.0008	H	-7.1507	-0.4997	0.0000
C	6.2418	-1.0945	-0.0002	H	-7.2762	-2.9854	0.0010
C	6.3108	-2.4884	-0.0008	H	-5.1916	-4.3286	0.0018
C	5.1357	-3.2441	-0.0013	H	-3.0081	-3.2643	0.0015
C	3.8819	-2.6276	-0.0011	H	-1.6077	2.5041	-0.0008
H	-5.5354	4.2178	-0.0015	H	-3.0696	4.4629	-0.0015
H	-6.5637	1.9528	-0.0009				

**T1^{+}**							
Element	Coordinates (angstroms)			Element	Coordinates (angstroms)		
	X	Y	Z		X	Y	Z
C	1.2732	-0.5526	-0.0019	C	-4.9507	-2.9733	1.3518
C	0.6892	-1.8446	-0.0095	C	-3.7315	-2.3702	1.0297
C	-0.6891	-1.8446	0.0112	C	-2.7100	2.1734	-1.0449
C	-1.2733	-0.5527	0.0023	C	-3.5644	3.2015	-1.4531
S	0.0001	0.6619	-0.0003	C	-4.9491	3.0628	-1.3400
C	2.6453	-0.2346	0.0278	C	-5.5153	1.8861	-0.8213
C	-2.6453	-0.2346	-0.0276	H	1.2807	-2.7481	0.0286
C	3.2603	1.0065	0.4976	H	-1.2805	-2.7482	-0.0262
C	4.6736	0.8688	0.4059	H	1.6416	2.2945	1.1760
C	4.9685	-0.4563	-0.1577	H	3.1461	4.1106	1.8711
C	3.7338	-1.1209	-0.3956	H	5.5985	3.8684	1.6676
C	-3.2601	1.0066	-0.4975	H	6.5940	1.7830	0.7557

C	-4.6734	0.8689	-0.4061	H	7.1172	-0.5504	-0.3027
C	-4.9686	-0.4563	0.1573	H	7.0951	-2.8108	-1.3338
C	-3.7339	-1.1209	0.3956	H	4.9550	-3.9439	-1.8365
C	2.7102	2.1732	1.0453	H	2.8096	-2.8664	-1.3085
C	3.5648	3.2013	1.4534	H	-7.1173	-0.5501	0.3016
C	4.9494	3.0626	1.3400	H	-7.0957	-2.8105	1.3328
C	5.5155	1.8859	0.8210	H	-4.9558	-3.9437	1.8363
C	6.1735	-1.0549	-0.4842	H	-2.8102	-2.8663	1.3091
C	6.1576	-2.3303	-1.0732	H	-1.6413	2.2948	-1.1754
C	4.9501	-2.9734	-1.3520	H	-3.1456	4.1108	-1.8706
C	3.7310	-2.3703	-1.0296	H	-5.5981	3.8686	-1.6676
C	-6.1737	-1.0547	0.4834	H	-6.5938	1.7831	-0.7561
C	-6.1580	-2.3300	1.0725				

$T1^{2+}$

Element	Coordinates (angstroms)			Element	Coordinates (angstroms)		
	X	Y	Z		X	Y	Z
C	1.2552	-0.5510	0.0064	C	-4.822	-2.591	1.989
C	0.7002	-1.8337	-0.0101	C	-3.631	-2.069	1.477
C	-0.7001	-1.8336	0.0096	C	-2.716	1.864	-1.607
C	-1.2552	-0.5509	-0.0067	C	-3.571	2.795	-2.204
S	0.0001	0.6662	-0.0003	C	-4.949	2.721	-1.991
C	2.6553	-0.2213	0.0609	C	-5.520	1.710	-1.181
C	-2.6553	-0.2212	-0.0609	H	1.299	-2.735	0.027
C	3.2676	0.8774	0.7721	H	-1.299	-2.734	-0.027
C	4.6809	0.7982	0.5816	H	1.652	1.908	1.810
C	4.9518	-0.3679	-0.2865	H	3.164	3.568	2.845
C	3.6978	-0.9755	-0.5960	H	5.601	3.445	2.469
C	-3.2677	0.8774	-0.7720	H	6.596	1.665	1.052
C	-4.6810	0.7981	-0.5815	H	7.086	-0.433	-0.581
C	-4.9517	-0.3680	0.2866	H	6.962	-2.420	-2.065
C	-3.6977	-0.9755	0.5961	H	4.795	-3.439	-2.663
C	2.7160	1.8640	1.6072	H	2.682	-2.494	-1.782
C	3.5708	2.7949	2.2038	H	-7.086	-0.434	0.581
C	4.9484	2.7213	1.9914	H	-6.962	-2.420	2.065
C	5.5199	1.7098	1.1810	H	-4.794	-3.440	2.664
C	6.1213	-0.8785	-0.8022	H	-2.682	-2.493	1.782
C	6.0460	-2.0098	-1.6521	H	-1.652	1.908	-1.810
C	4.8222	-2.5911	-1.9889	H	-3.165	3.569	-2.845
C	3.6316	-2.0690	-1.4765	H	-5.602	3.445	-2.468
C	-6.1211	-0.8789	0.8024	H	-6.597	1.665	-1.052
C	-6.0457	-2.0101	1.6523				

T2

Element	Coordinates (angstroms)			Element	Coordinates (angstroms)		
	X	Y	Z		X	Y	Z

C	-3.2299	0.3585	-0.0486	C	7.6034	1.9186	0.4388
C	-2.5355	1.6169	-0.0013	C	7.1251	3.2143	0.6505
C	-1.1737	1.5327	0.0064	C	5.7506	3.4691	0.6328
C	-0.6599	0.2016	-0.0344	C	4.8314	2.4422	0.4027
S	-2.0119	-0.9458	-0.0805	C	5.6139	-2.5092	-0.5843
C	0.6599	-0.2016	-0.0344	C	6.8069	-3.2270	-0.6991
S	2.0119	0.9458	-0.0805	C	8.0422	-2.6096	-0.4816
C	3.2299	-0.3585	-0.0486	C	8.1000	-1.2499	-0.1685
C	2.5355	-1.6169	-0.0012	H	-3.0637	2.5533	0.0953
C	1.1737	-1.5327	0.0064	H	-0.5196	2.3951	0.0697
C	-4.5934	0.1357	-0.0481	H	3.0637	-2.5533	0.0954
C	4.5934	-0.1357	-0.0481	H	0.5196	-2.3951	0.0698
C	-5.2946	-1.1395	0.1795	H	-3.7746	-2.6785	0.4072
C	-6.6939	-0.8921	0.2045	H	-5.3879	-4.4787	0.8007
C	-6.9142	0.5298	-0.0523	H	-7.8233	-4.0261	0.8314
C	-5.6502	1.1553	-0.2279	H	-8.6709	-1.7183	0.4584
C	5.6502	-1.1553	-0.2279	H	-9.0593	0.7596	-0.0288
C	6.9142	-0.5298	-0.0523	H	-8.9589	3.1840	-0.5752
C	6.6939	0.8921	0.2045	H	-6.7700	4.2773	-0.9724
C	5.2946	1.1395	0.1795	H	-4.6837	3.0134	-0.8133
C	-4.8314	-2.4422	0.4027	H	8.6709	1.7183	0.4584
C	-5.7506	-3.4691	0.6328	H	7.8233	4.0261	0.8314
C	-7.1251	-3.2143	0.6505	H	5.3879	4.4787	0.8007
C	-7.6034	-1.9186	0.4388	H	3.7746	2.6785	0.4072
C	-8.1000	1.2499	-0.1685	H	4.6837	-3.0134	-0.8133
C	-8.0422	2.6096	-0.4816	H	6.7700	-4.2773	-0.9724
C	-6.8069	3.2270	-0.6991	H	8.9589	-3.1841	-0.5752
C	-5.6139	2.5092	-0.5843	H	9.0593	-0.7596	-0.0289

$T2^{+}$

Element	Coordinates (angstroms)			Element	Coordinates (angstroms)		
	X	Y	Z		X	Y	Z
C	-3.2072	0.3697	0.0092	C	7.6144	1.8397	0.5639
C	-2.5408	1.6198	0.0819	C	7.1414	3.0905	0.9894
C	-1.1638	1.5349	0.1189	C	5.7712	3.3348	1.1028
C	-0.6728	0.2133	0.0667	C	4.8410	2.3399	0.7888
S	-2.0023	-0.9262	-0.0153	C	5.5199	-2.4134	-0.9277
C	0.6728	-0.2132	0.0667	C	6.6917	-3.1195	-1.2151
S	2.0023	0.9262	-0.0152	C	7.9437	-2.5407	-0.9979
C	3.2072	-0.3697	0.0092	C	8.0514	-1.2299	-0.5089
C	2.5407	-1.6197	0.0818	H	-3.0780	2.5521	0.1807
C	1.1637	-1.5348	0.1188	H	-0.5125	2.3967	0.2068
C	-4.5901	0.1372	-0.0189	H	3.0780	-2.5521	0.1806
C	4.5900	-0.1371	-0.0190	H	0.5125	-2.3966	0.2067
C	-5.2981	-1.0950	0.3389	H	-3.7854	-2.5507	0.9124
C	-6.6963	-0.8530	0.2427	H	-5.4224	-4.3037	1.4444

C	-6.8923	0.5281	-0.2175	H	-7.8495	-3.8734	1.2417
C	-5.6135	1.1235	-0.3911	H	-8.6816	-1.6548	0.4947
C	5.6134	-1.1235	-0.3911	H	-9.0283	0.7758	-0.3752
C	6.8923	-0.5281	-0.2175	H	-8.8435	3.1015	-1.2303
C	6.6963	0.8529	0.2427	H	-6.6247	4.1216	-1.6252
C	5.2982	1.0950	0.3389	H	-4.5642	2.8657	-1.1615
C	-4.8409	-2.3399	0.7888	H	8.6817	1.6546	0.4946
C	-5.7710	-3.3348	1.1028	H	7.8497	3.8733	1.2416
C	-7.1413	-3.0906	0.9894	H	5.4226	4.3037	1.4444
C	-7.6143	-1.8398	0.5640	H	3.7855	2.5508	0.9124
C	-8.0514	1.2298	-0.5089	H	4.5640	-2.8657	-1.1615
C	-7.9438	2.5406	-0.9979	H	6.6245	-4.1217	-1.6251
C	-6.6918	3.1195	-1.2151	H	8.8433	-3.1016	-1.2303
C	-5.5200	2.4134	-0.9277	H	9.0283	-0.7760	-0.3753

T2^{2+}

Element	Coordinates (angstroms)			Element	Coordinates (angstroms)		
	X	Y	Z		X	Y	Z
C	-3.1930	0.3866	0.0549	C	7.6396	1.7171	0.7128
C	-2.5470	1.6288	0.1381	C	7.1807	2.8801	1.3698
C	-1.1563	1.5379	0.2096	C	5.8220	3.0882	1.6119
C	-0.6842	0.2241	0.1582	C	4.8783	2.1439	1.1980
S	-2.0041	-0.9097	0.0572	C	5.4048	-2.2639	-1.2838
C	0.6842	-0.2241	0.1582	C	6.5406	-2.9327	-1.7494
S	2.0041	0.9097	0.0572	C	7.8112	-2.3966	-1.5348
C	3.1930	-0.3866	0.0549	C	7.9852	-1.1657	-0.8634
C	2.5470	-1.6288	0.1381	H	-3.0873	2.5628	0.2176
C	1.1563	-1.5379	0.2096	H	-0.5071	2.4002	0.3068
C	-4.5963	0.1513	0.0027	H	3.0873	-2.5628	0.2176
C	4.5963	-0.1513	0.0027	H	0.5071	-2.4002	0.3068
C	-5.3181	-1.0021	0.5108	H	-3.8314	-2.3024	1.4293
C	-6.7106	-0.7898	0.2908	H	-5.4977	-3.9802	2.1360
C	-6.8677	0.5093	-0.3929	H	-7.9027	-3.6176	1.7056
C	-5.5690	1.0678	-0.5691	H	-8.7028	-1.5667	0.5572
C	5.5690	-1.0678	-0.5691	H	-8.9810	0.7534	-0.7383
C	6.8677	-0.5093	-0.3929	H	-8.6835	2.9222	-1.9102
C	6.7106	0.7898	0.2908	H	-6.4324	3.8628	-2.2960
C	5.3181	1.0021	0.5108	H	-4.4218	2.6608	-1.5088
C	-4.8784	-2.1439	1.1980	H	8.7028	1.5667	0.5572
C	-5.8220	-3.0882	1.6119	H	7.9026	3.6176	1.7056
C	-7.1807	-2.8800	1.3697	H	5.4977	3.9802	2.1360
C	-7.6396	-1.7170	0.7128	H	3.8313	2.3024	1.4293
C	-7.9852	1.1657	-0.8634	H	4.4218	-2.6608	-1.5088
C	-7.8112	2.3966	-1.5348	H	6.4324	-3.8628	-2.2960
C	-6.5406	2.9328	-1.7493	H	8.6835	-2.9221	-1.9102
C	-5.4047	2.2639	-1.2838	H	8.9810	-0.7534	-0.7383

5. Synthetic Details and NMR spectra

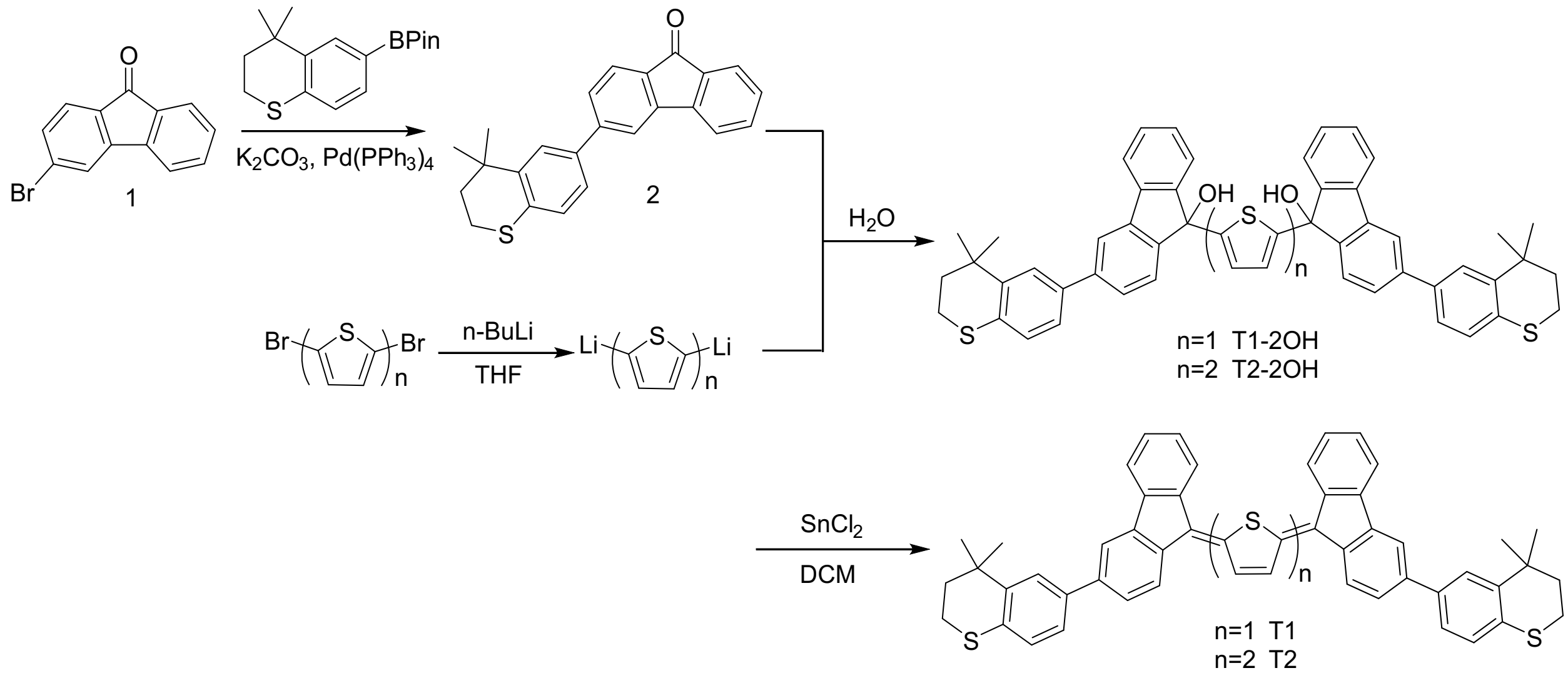

3-(4,4-dimethylthiochroman-6-yl)-9H-fluoren-9-one (**2**)

3-bromo-9H-fluoren-9-one (**1**) 1.5 g 5.8 mmol and 2-(4,4- dimethylthiochroman-6-yl)-4,4,5,5-tetramethyl-1,3,2-dioxaborolane (Thiocroman-Bpin) 2.1 g 6.9 mmol (1.2 eqv.) were added into a 50 mL schlenk flask, followed by the addition of 10 mL THF/H_2O = 4:1. The mixture was purged with N_2 for 20 min, and then, a suspension of K_2CO_3 (2.4g 17.4 mmol) in 1 mL water was added. While stirring the mixture under nitrogen for another 10 min, $Pd(PPh_3)_4$ (334 mg, 0.29 mmol) was added in one portion, and the mixture was heated to reflux for 12h. The reaction was monitored by thin-layer chromatography (TLC). After the starting materials were consumed, the mixture was cooled to room temperature. The mixture was diluted with 50 mL of dichloromethane and washed with DI-water (100 mL×3). The organic phase was dried with $MgSO_4$, filtered, and the solvent was removed under vacuum. The crude product was further purified by silica column to obtain the pure product compounds **2** as a light yellow solid (1.34 g 65%).

^{1}H NMR (400 MHz, Chloroform-*d*) δ 7.72 – 7.66 (m, 3H), 7.64 – 7.58 (m, 2H), 7.54 – 7.44 (m, 2H), 7.36 – 7.30 (m, 2H), 7.20 (d, *J* = 8.2 Hz, 1H), 3.14 – 3.04 (m, 2H), 2.08 – 1.97 (m, 2H), 1.42 (s, 6H).

^{13}C NMR (126 MHz, Chloroform-*d*) δ 193.38, 147.91, 145.23, 144.13, 142.56, 136, 134.83, 134.55, 133.03, 132.67, 129.21, 127.45, 127.16, 125.16, 124.79, 124.25, 120.29, 118.72, 37.52, 33.20, 30.21, 23.19

HRMS (ESI, pos.): m/z calcd for $C_{24}H_{20}OS$ $[M+H]^+$: 357.1313; found: 357.1313

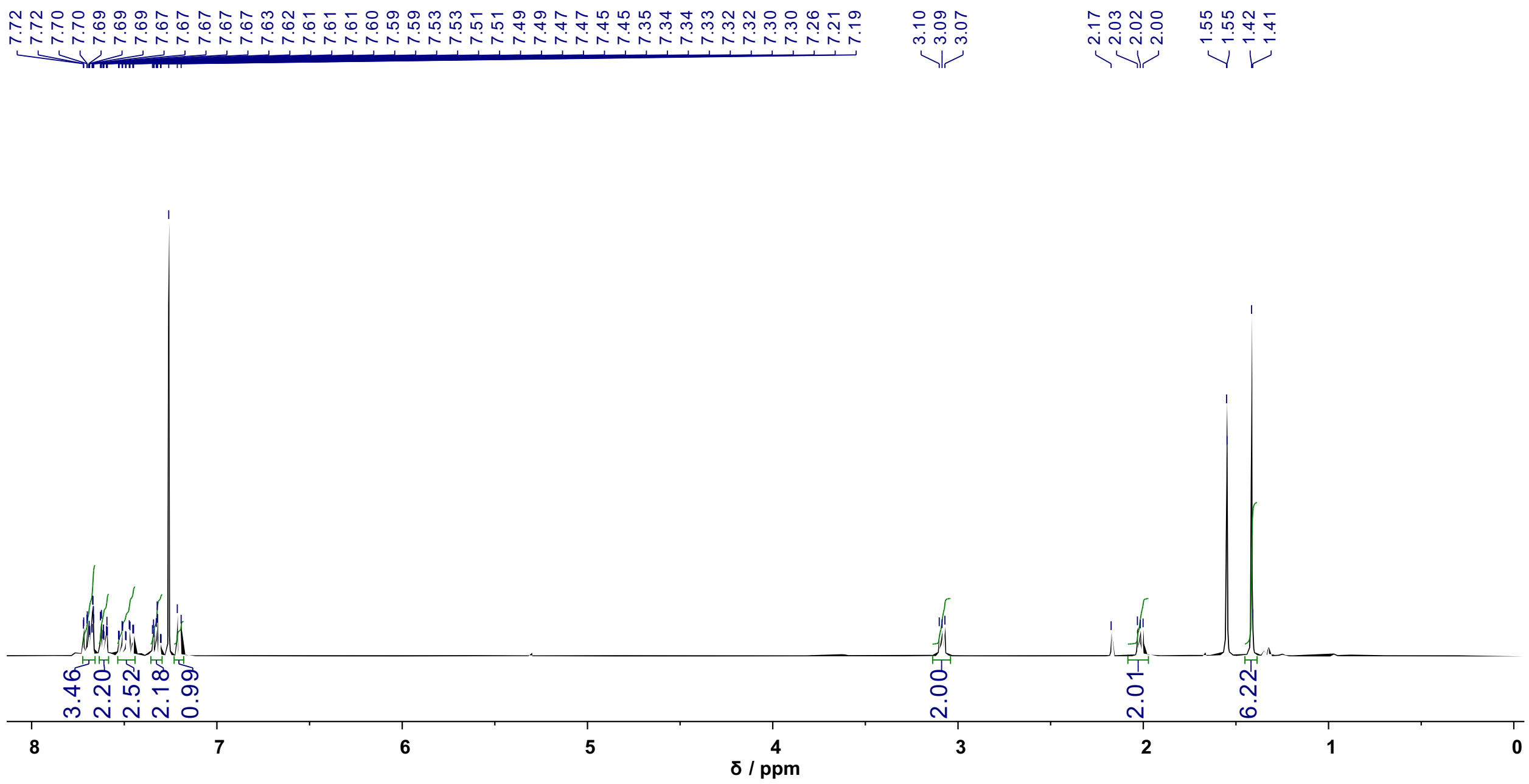

^{1}H NMR of compound **2**

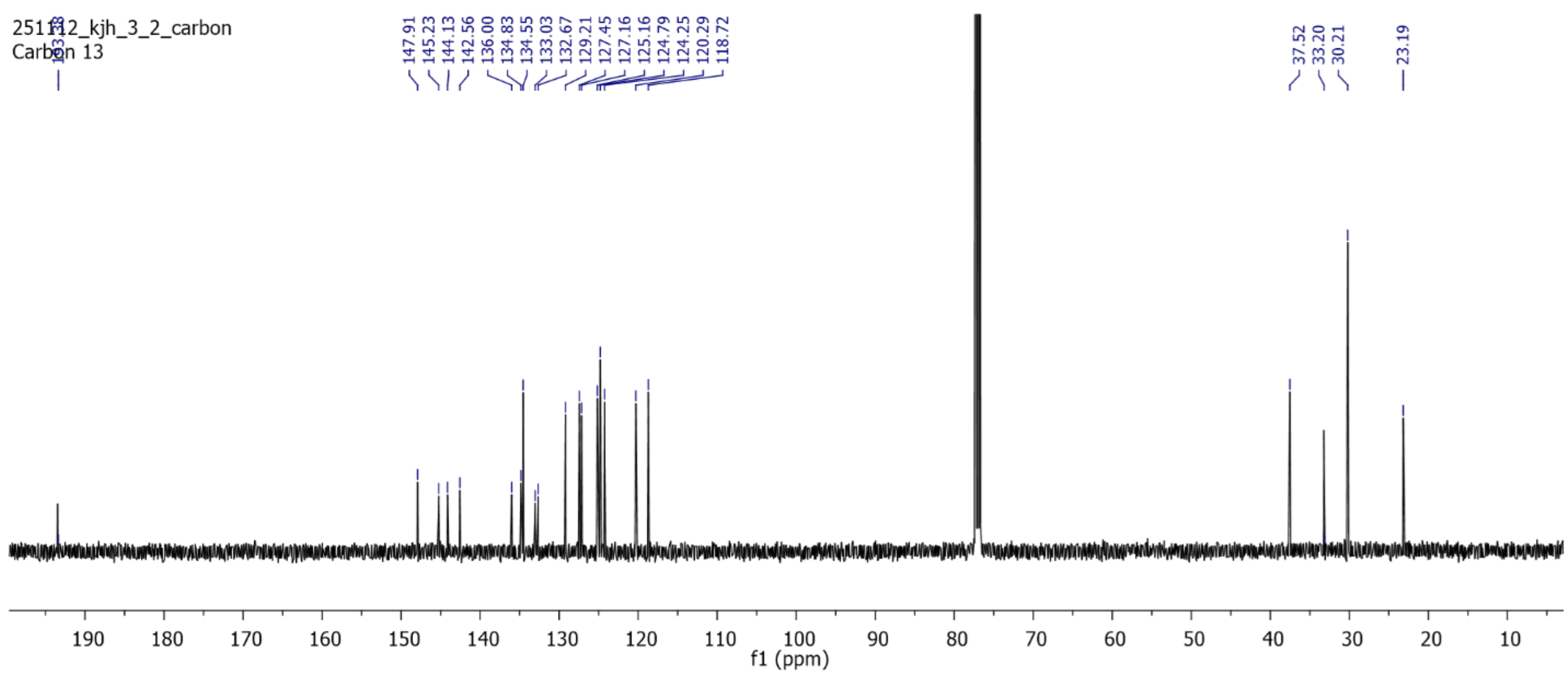

[1]C NMR of compound **2**

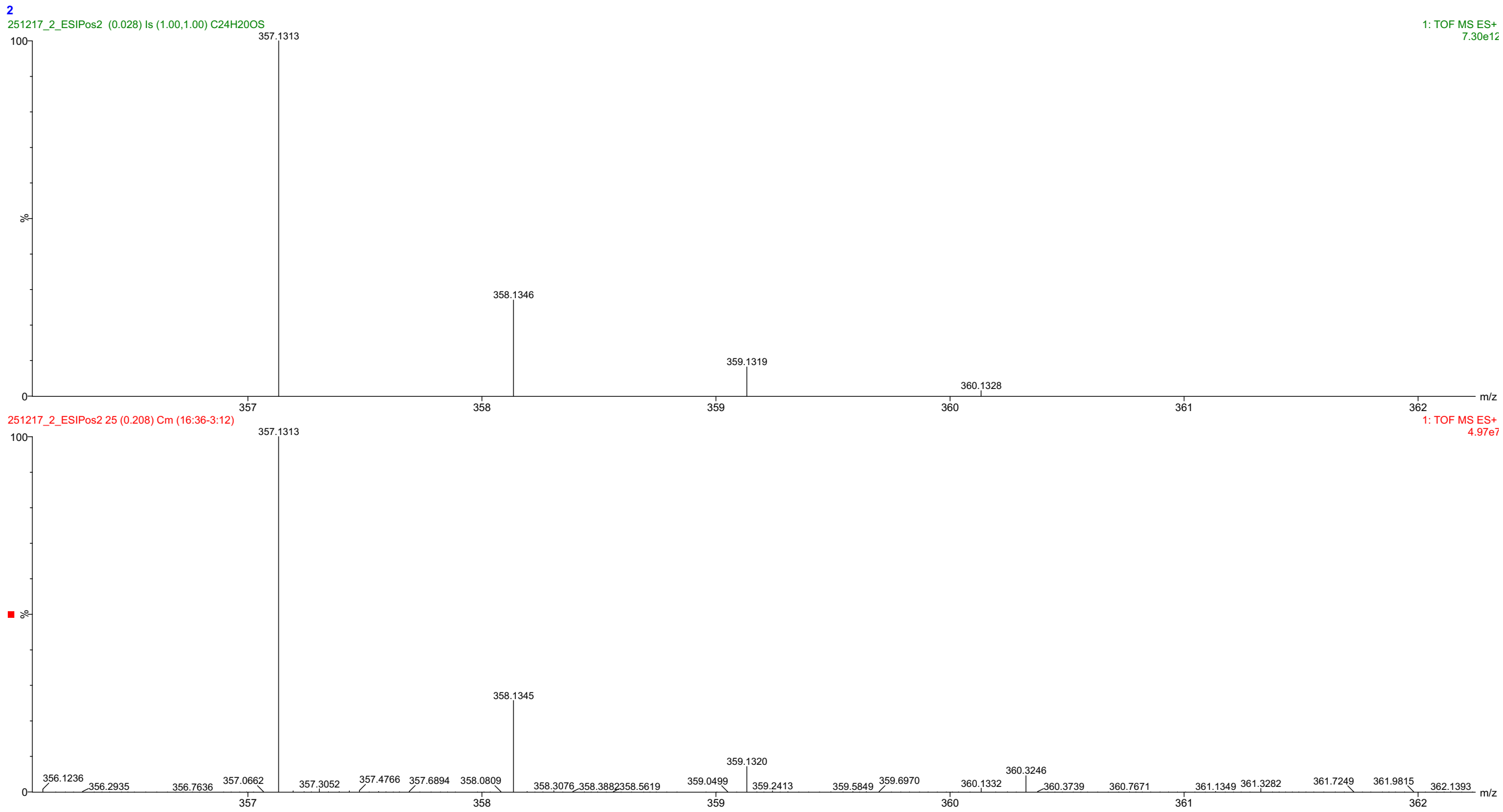

HRMS of compound **2** (ESI, pos.)

9,9'-(thiophene-2,5-diyl)bis(3-(4,4-dimethylthiochroman-6-yl)-9H-fluoren-9-ol) (**T1-OH**)

2,5-dibromothiophene (200 mg, 0.83 mmol) was charged into a 50 mL Schlenk flask. After three cycles of vacuum-heat purging under nitrogen atmosphere, 20 mL of anhydrous tetrahydrofuran (THF) was introduced via syringe under continuous nitrogen flow. The reaction system was subsequently cooled to -78 °C using a dry ice/acetone bath. A solution of n-BuLi (1.0 M in hexane, 2.1 mL, 2.1 mmol) was then added dropwise via a syringe over 10 minutes. The resulting mixture was maintained at -78 °C for 30 minutes, followed by gradual warming to room temperature over 1 hour with continuous stirring. The reaction vessel was recooled to -78 °C. A pre-cooled THF solution (1 mL) containing **2** (740 mg, 2.1 mmol) was introduced via syringe. The reaction mixture was allowed to warm gradually to ambient temperature and stirred for 12 hours under inert atmosphere. The reaction was quenched by careful addition of methanol/water (1:1 v/v, 5 mL), followed by dilution with dichloromethane (50 mL). The organic layer was sequentially washed with DI water (3 × 100 mL), dried over anhydrous magnesium sulfate, and filtered through a short pad of celite. Solvent removal under reduced pressure afforded the crude product, which was subsequently purified by silica column to yield the desired compound 9,9'-(thiophene-2,5-diyl)bis(3-(4,4-dimethylthiochroman-6-yl)-9H-fluoren-9-ol) (**T1-OH**) as a light yellow crystalline solid (330 mg, 50% yield).

^{1}H NMR (500 MHz, Acetone-d_6) δ 8.01 (d, J = 1.6 Hz, 2H), 7.85 (d, J = 7.5 Hz, 2H), 7.79 (d, J = 2.0 Hz, 2H), 7.62 – 7.51 (m, 6H), 7.40 (ddd, J = 15.3, 7.8, 1.6 Hz, 4H), 7.33 (d, J = 7.4 Hz, 2H), 7.14 (d, J = 8.2 Hz, 2H), 6.53 (s, 2H), 3.14 – 3.05 (m, 4H), 2.03 – 1.98 (m, 4H), 1.42 (s, 12H).
^{13}C NMR (126 MHz, Acetone) δ 151.32, 149.67, 149.46, 143.42, 142.82, 140.80, 137.60, 132.44, 129.78, 128.97, 127.70, 127.39, 126.21, 126.01, 125.75, 125.55, 123.74, 121.12, 119.07, 82.44, 38.59, 33.90, 23.48.
HRMS (ESI, pos.): m/z calcd for $C_{52}H_{44}O_2S_3$ [M+Na]$^+$: 819.2401; found: 819.2388

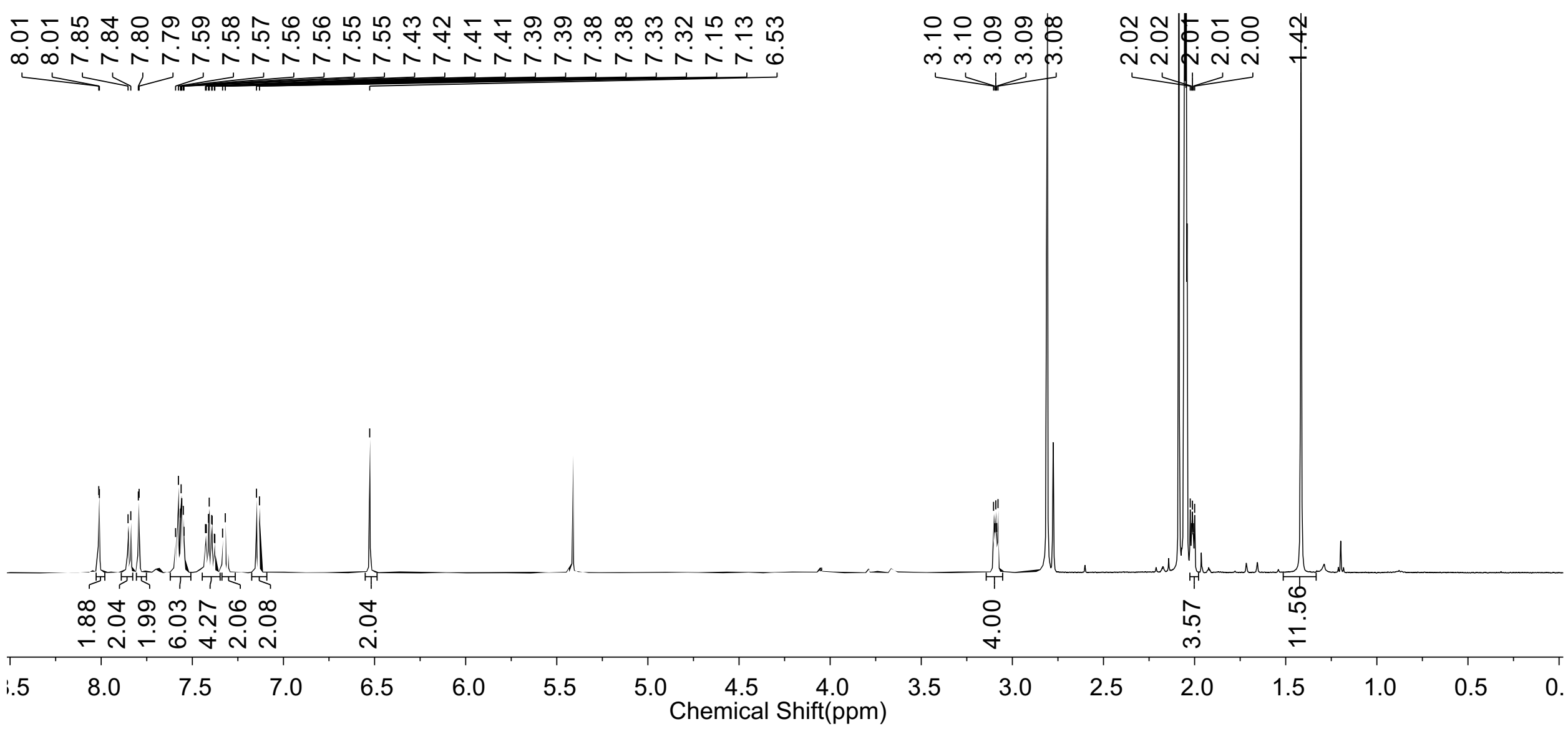

^{1}H NMR of **T1-OH** (500 MHz, Acetone-d_6)

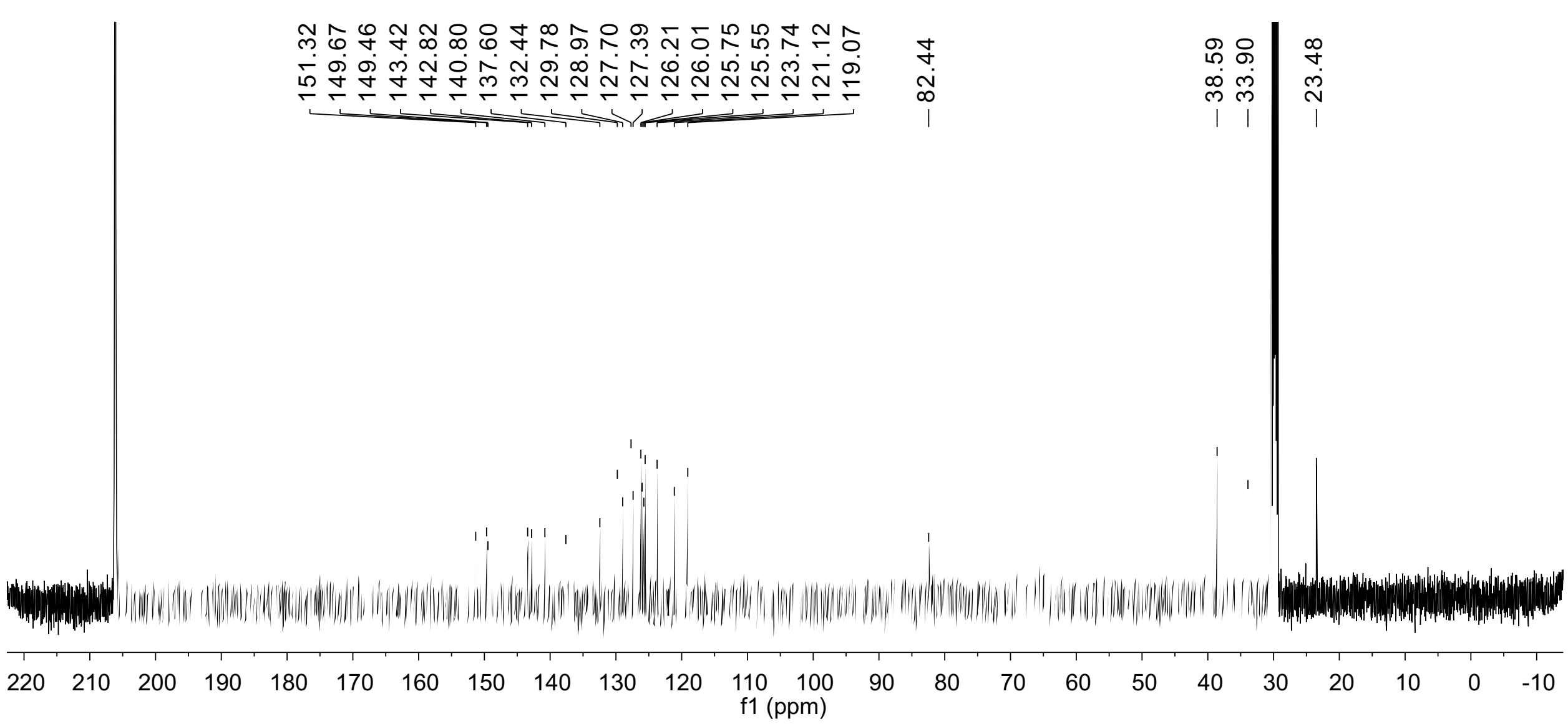

^{13}C NMR of **T1-OH** (126 MHz, Acetone-d$_6$)

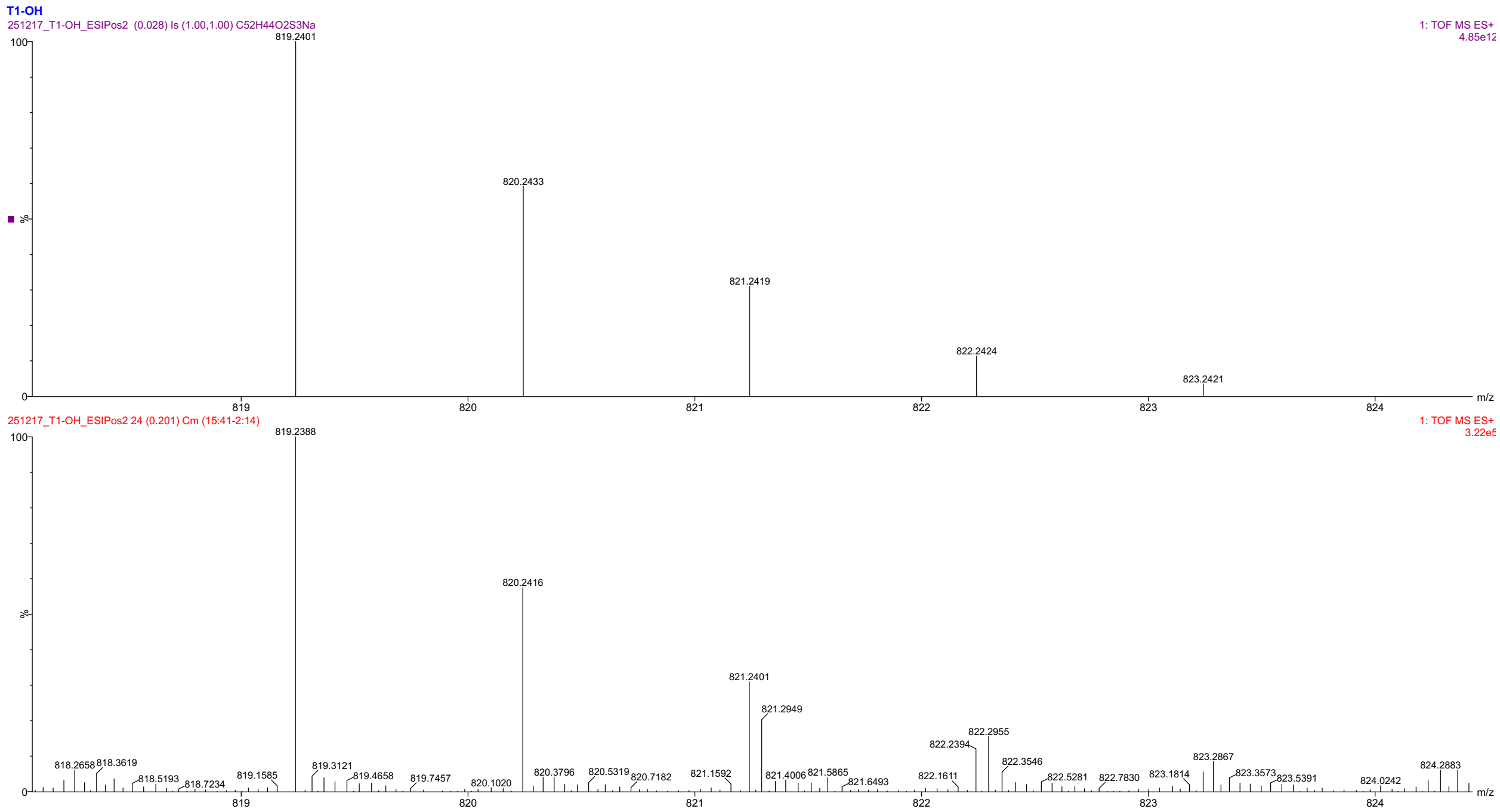

HRMS of compound **T1-OH** (ESI, pos.)

3-(4,4-dimethylthiochroman-6-yl)-9-(5'-(3-(4,4-dimethylthiochroman-6-yl)-9-hydroxy-9H-fluoren-9-yl)-[2,2'-bithiophen]-5-yl)-9H-fluoren-9-ol (**T2-OH**)

5,5'-dibromo-2,2'-bithiophene (300 mg, 0.93 mmol) was charged into a 50 mL Schlenk flask. After three cycles of vacuum-heat purging under nitrogen atmosphere, 20 mL of anhydrous tetrahydrofuran (THF) was introduced via syringe under continuous nitrogen flow. The reaction system was subsequently cooled to -78 °C using a dry ice/acetone bath. A solution of n-BuLi (1.0 M in hexane, 2.3 mL, 2.3 mmol) was then added dropwise via a syringe over 10 minutes. The resulting mixture was maintained at -78 °C for 30 minutes, followed by gradual warming to room temperature over 1 hour with continuous stirring. The reaction vessel was recooled to -78 °C. A pre-cooled THF solution (1 mL) containing **2** (830 mg, 2.3 mmol) was introduced via syringe. The reaction mixture was allowed to warm gradually to ambient temperature and stirred for 12 hours under inert atmosphere. The reaction was quenched by careful addition of methanol/water (1:1 v/v, 5 mL), followed by dilution with dichloromethane (50 mL). The organic layer was sequentially washed with DI water (3 × 100 mL), dried over anhydrous magnesium sulfate, and filtered through a short pad of celite. Solvent removal under reduced pressure afforded the crude product, which was subsequently purified by silica column to yield the desired compound 3-(4,4-

dimethylthiochroman-6-yl)-9-(5'-(3-(4,4-dimethylthiochroman-6-yl)-9-hydroxy-9H-fluoren-9-yl)-[2,2'-bithiophen]-5-yl)-9H-fluoren-9-ol (**T2-OH**) as a light yellow crystalline solid (433 mg, 53% yield).

^{1}H NMR (500 MHz, Acetone-d$_6$) δ 8.04 (d, J = 1.7 Hz, 2H), 7.87 (dt, J = 7.5, 0.9 Hz, 2H), 7.81 (d, J = 2.0 Hz, 2H), 7.63 – 7.55 (m, 6H), 7.45 – 7.39 (m, 4H), 7.34 (td, J = 7.4, 1.1 Hz, 2H), 7.15 (d, J = 8.2 Hz, 2H), 6.97 (d, J = 3.7 Hz, 2H), 6.64 (d, J = 3.8 Hz, 2H), 3.13 – 3.05 (m, 4H), 2.01 (d, J = 6.0 Hz, 4H), 1.42 (s, 12H).

^{13}C NMR (126 MHz, Acetone-d$_6$) δ151.17, 151.14, 149.98, 149.50, 143.54, 143.11, 140.93, 140.12, 137.66, 137.36, 132.61, 130.06, 129.22, 127.83, 127.63, 126.31, 126.05, 125.77, 125.67, 125.22, 123.78, 121.32, 119.26, 82.47, 60.65, 38.68, 34.00, 23.59, 20.93, 14.61.

HRMS (ESI, pos.): m/z calcd for $C_{56}H_{46}O_2S_4$ [M+Na]$^+$: 901.2278; found: 901.2274

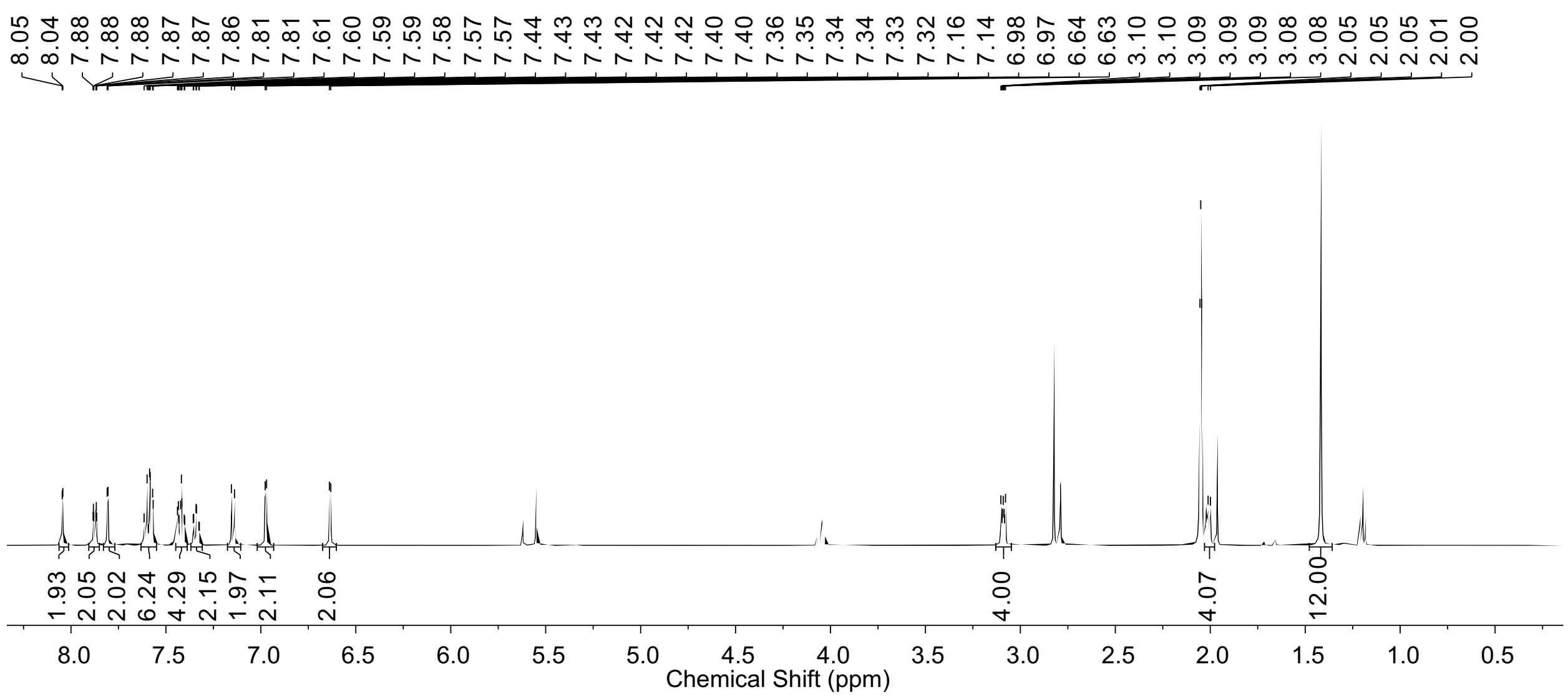

^{1}H NMR of **T2-OH** (500 MHz, Acetone-d$_6$)

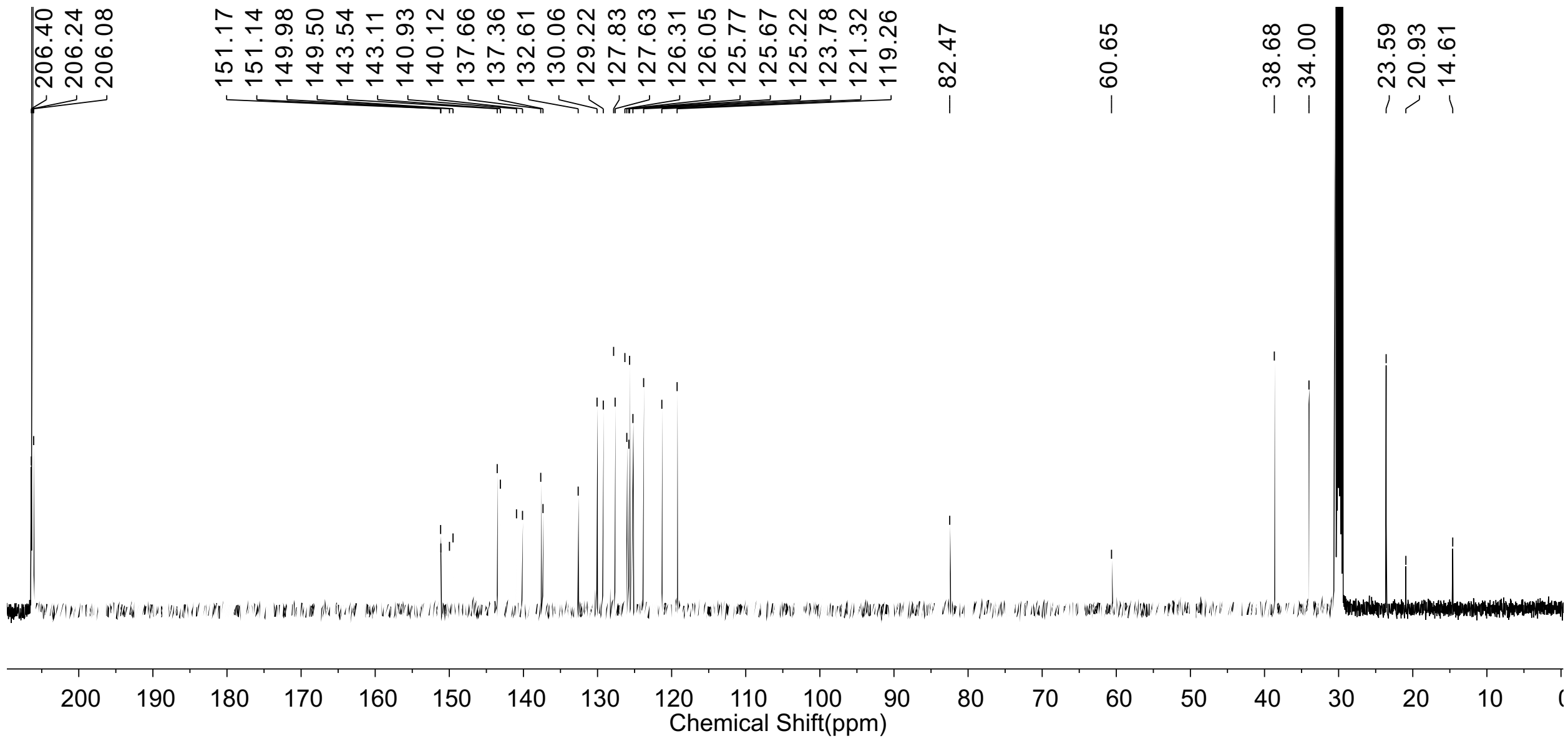

^{13}C NMR of **T2-OH** (126 MHz, Acetone-d$_6$)

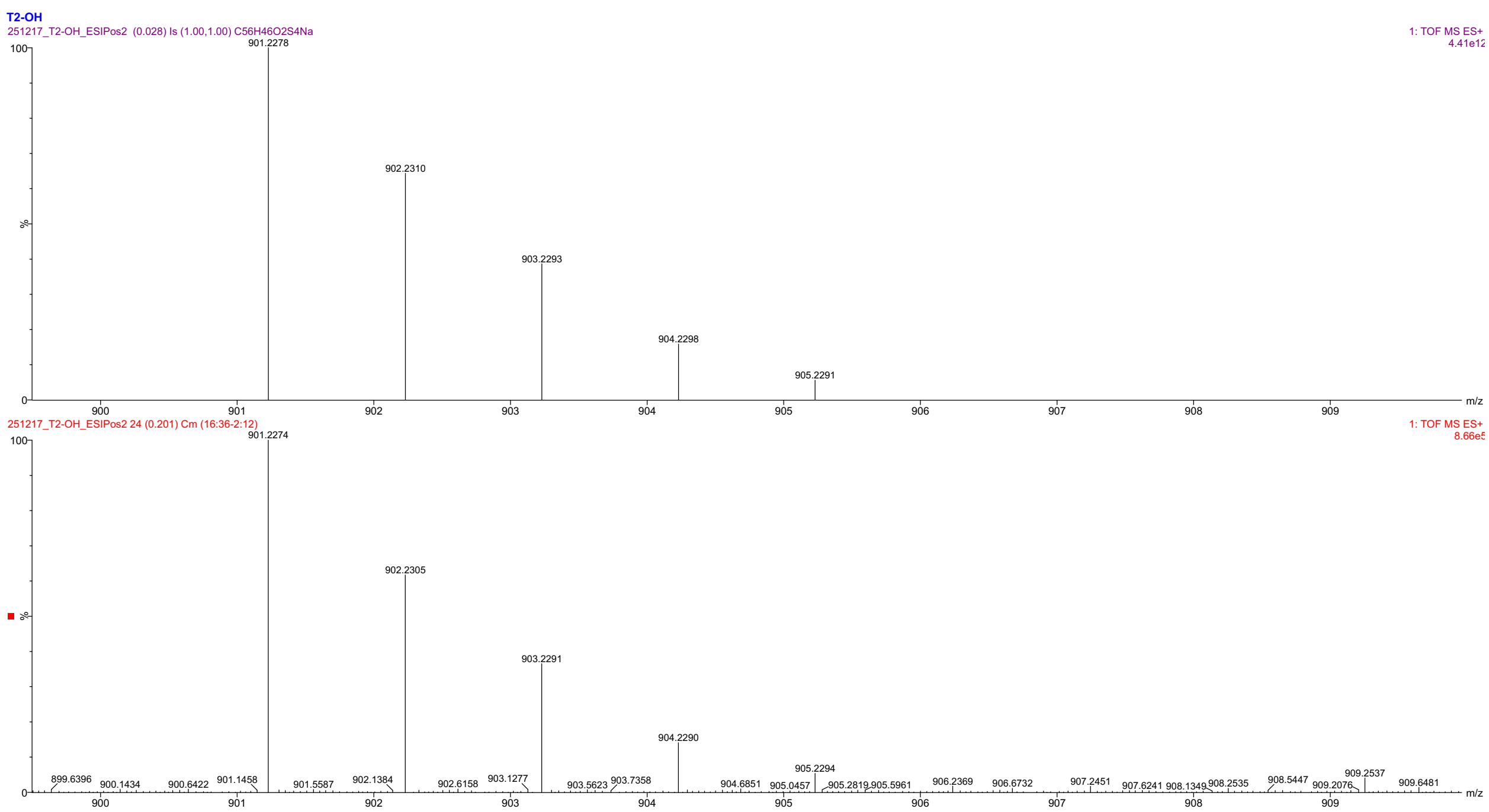

HRMS of compound **T2-OH** (ESI, pos.)

2,5-bis(3-(4,4-dimethylthiochroman-6-yl)-9H-fluoren-9-ylidene)-2,5-dihydrothiophene (**T1**)/ (5-(3-(4,4-dimethylthiochroman-6-yl)-9H-fluoren-9-ylidene)-5'-(3-(4,4-dimethylthiochroman-6-yl)-9H-fluoren-9-ylidene)-5H,5'H-2,2'-bithiophenylidene (**T2**)

T1-OH (150 mg, 0.19 mmol) or **T2-OH** (150 mg, 0.17 mmol) was charged into a 50 mL Schlenk flask. After three cycles of vacuum-heat purging under nitrogen atmosphere, 20 mL of anhydrous

Dichloromethane (DCM) was introduced via syringe under continuous nitrogen flow. 190.0 mg 1 mmol of Tin (II) chloride ($SnCl_2$) was added into the flask in one portion under N_2, then the mixture was stirred for 1h and monitored by TLC plate. After the starting materials were consumed, the mixture was cooled to room temperature. The mixture was diluted with 50 mL of dichloromethane and washed with DI-water (100 mL×3). The organic phase was dried with $MgSO_4$, filtered, and the solvent was removed under vacuum. The crude product was further purified by silica column to obtain the pure product as purple solid for 2,5-bis(3-(4,4-dimethylthiochroman-6-yl)-9H-fluoren-9-ylidene)-2,5-dihydrothiophene (**T1**) (72 mg, 50%) and deep blue solid for (5-(3-(4,4-dimethylthiochroman-6-yl)-9H-fluoren-9-ylidene)-5'-(3-(4,4-dimethylthiochroman-6-yl)-9H-fluoren-9-ylidene)-5H,5'H-2,2'-bithiophenylidene (**T2**) (86 mg, 60%).

T1 ^{1}H NMR (400 MHz, Tetrachloroethane-*d*$_2$) δ 8.36 – 8.22 (m, 4H), 8.09 (d, *J* = 8.4 Hz, 2H), 7.97 (d, *J* = 5.6 Hz, 2H), 7.90 (d, *J* = 7.5 Hz, 2H), 7.76 – 7.69 (m, 2H), 7.65 – 7.39 (m, 8H), 7.22 (d, *J* = 8.3 Hz, 2H), 3.19 – 2.99 (m, 4H), 2.11 – 1.98 (m, 4H), 1.45 (s, 12H).

^{13}C NMR(500 MHz, Chloroform-*d*) δ 146.08,144.32, 142.39, 140.70, 139.90, 138.17, 137.06, 129.14, 127.99, 127.03, 126.67, 125.35, 125.18, 124.94, 120.06, 118.38,75.10, 37.72, 33.22, 30.29, 29.70, 23.16, 14.11.

HRMS (MALDI-TOF, pos.): m/z calcd for $C_{52}H_{42}S_3$ $[M]^+$: 762.2449; found: 762.3004.

T2 ^{1}H NMR (400 MHz, Chloroform-*d*) δ 8.16 (dd, *J* = 12.3, 8.0 Hz, 1H), 8.07 (s, 1H), 7.93 (dd, *J* = 19.3, 6.0 Hz, 5H), 7.83 (d, *J* = 7.0 Hz, 2H), 7.75 – 7.67 (m, 2H), 7.64 (d, *J* = 8.5 Hz, 1H), 7.55 – 7.30 (m, 9H), 7.21 (dd, *J* = 8.2, 5.8 Hz, 2H), 3.20 – 2.99 (m, 4H), 2.04 (dd, *J* = 5.8, 3.2 Hz, 4H), 1.44 (d, *J* = 2.5 Hz, 11H).

^{1}H NMR (500 MHz, Acetone-*d*$_6$) δ 8.49 (s, 2H), 8.43 – 8.31 (m, 2H), 8.29 – 8.17 (m, 4H), 8.04 (d, *J* = 9.0 Hz, 2H), 7.97 – 7.86 (m, 2H), 7.79 (t, *J* = 8.5 Hz, 1H), 7.67 (d, *J* = 8.2 Hz, 1H), 7.60 – 7.35 (m, 7H), 7.19 (t, *J* = 7.8 Hz, 2H), 3.12 (dt, *J* = 9.4, 3.6 Hz, 4H), 1.46 (d, *J* = 9.2 Hz, 12H). 4H at ca. 2 ppm is overlapped by the solvent peak.

^{13}C NMR(500 MHz, Chloroform-*d*) δ 147.91, 145.24, 144.13, 142.61, 142.56, 142.43, 134.83, 134.55, 133.04, 132.67, 129.21, 129.04, 128.23, 127.45, 127.16, 127.05, 126.97, 125.16, 124.93, 124.79, 124.77, 124.25, 120.29, 118.71, 67.98, 53.41, 37.73, 37.52, 33.28, 33.23, 30.27, 30.20, 29.7, 25.61, 23.18, 22.69, 14.11

HRMS (MALDI-TOF, pos.): m/z calcd for $C_{56}H_{44}S_4$ $[M]^+$: 844.2326; found: 844.2058.

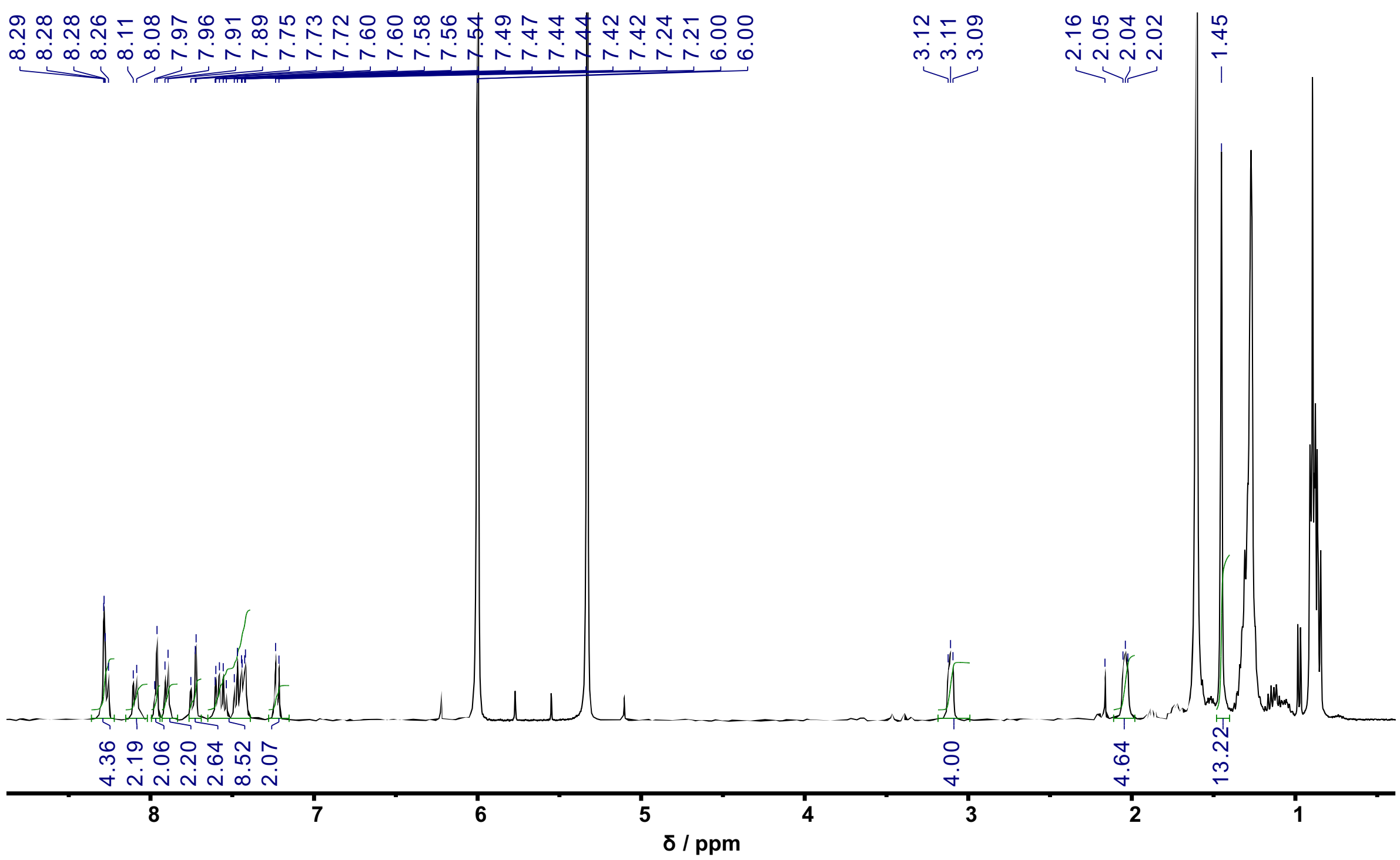

^{1}H NMR of **T1** (400 MHz, Tetrachloroethane-d_2)

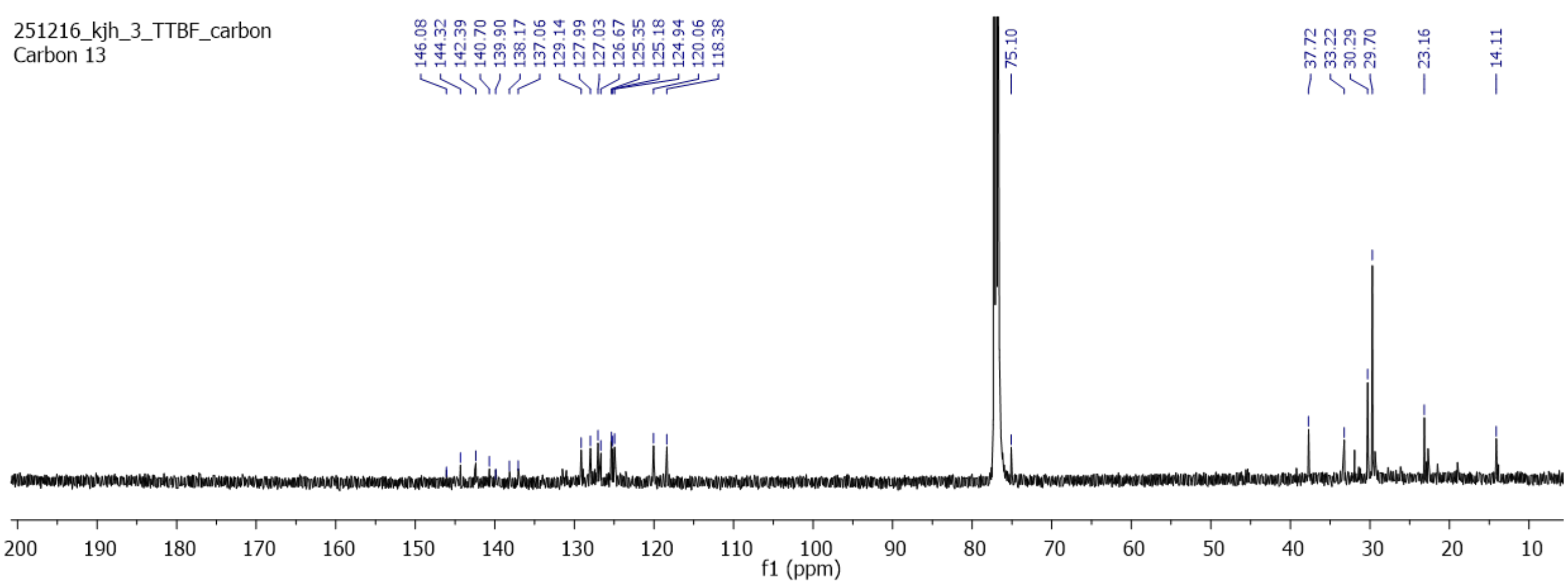

^{13}C NMR of **T1** (500 MHz, Chloroform-d)

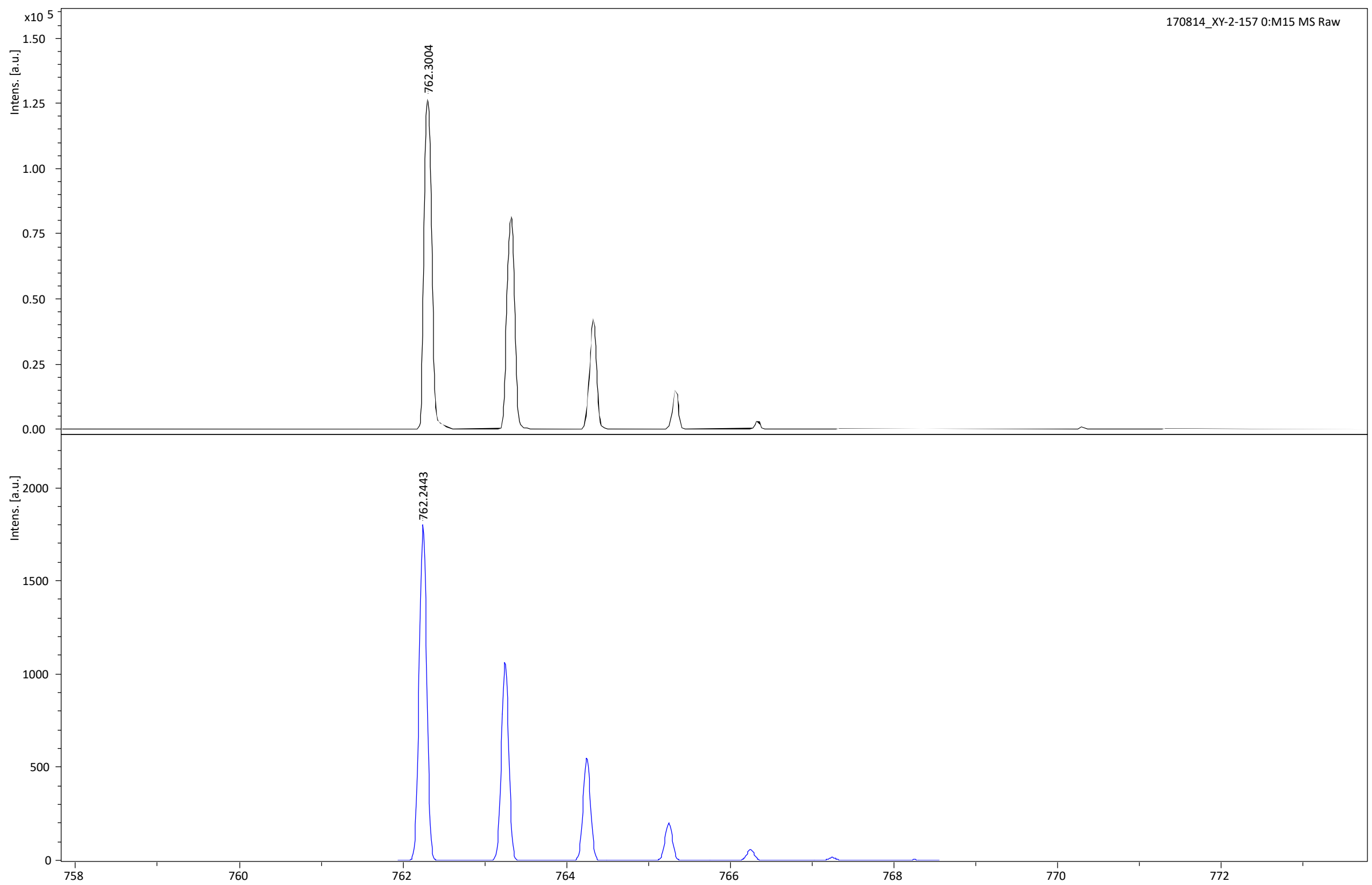

MALDI of **T1**

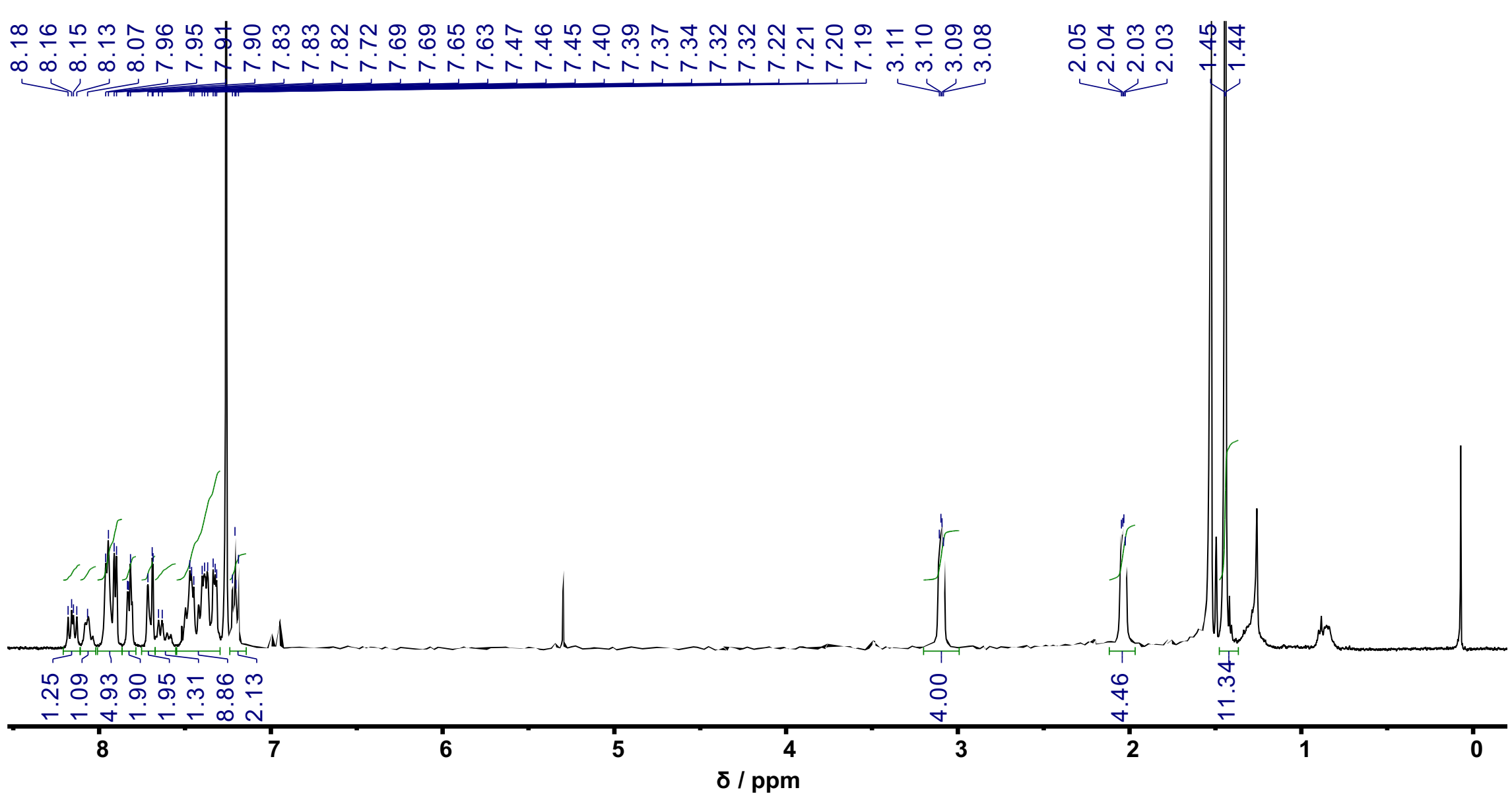

^{1}H NMR of **T2** (400 MHz, Chloroform-*d*)

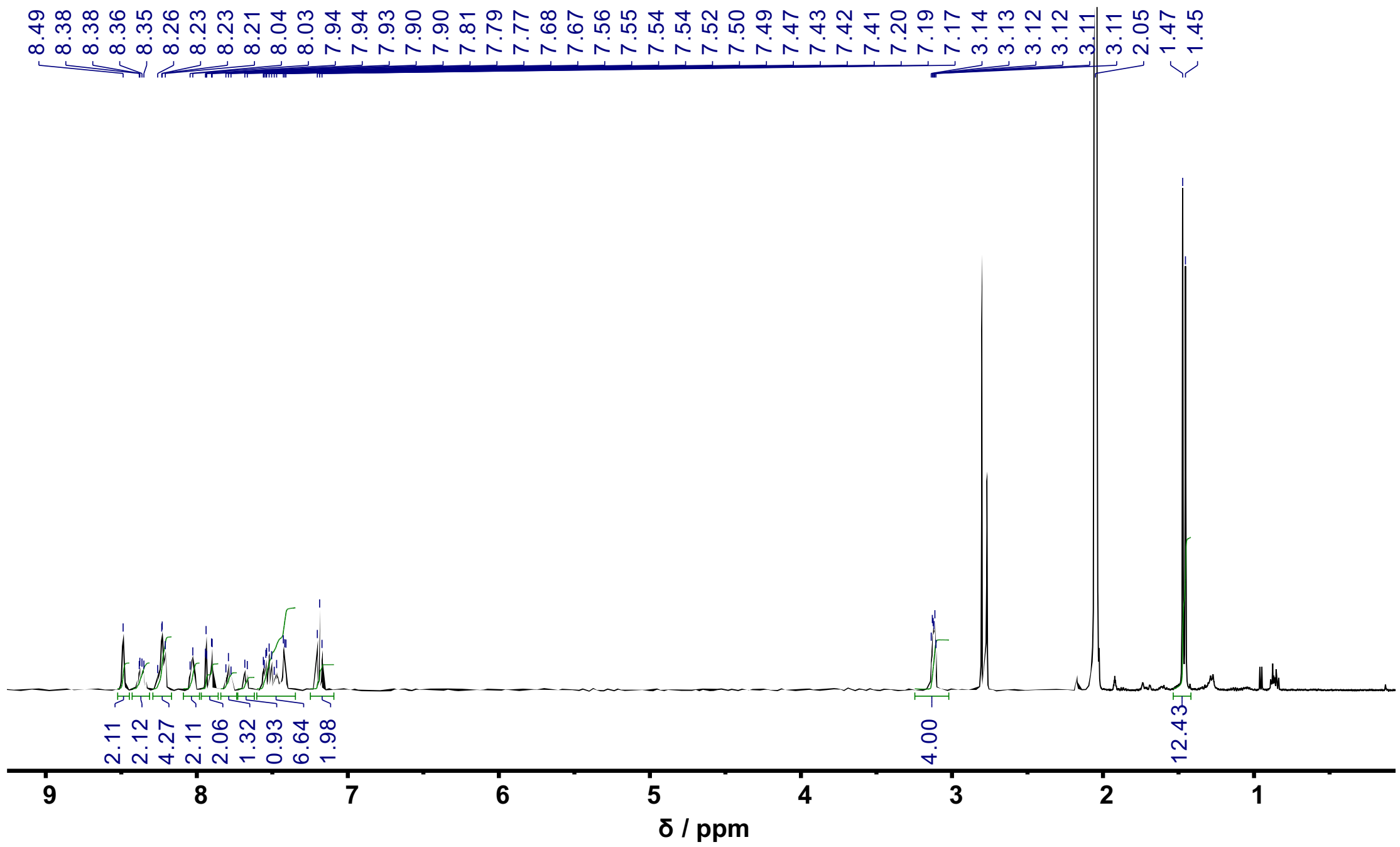

^{1}H NMR of **T2** (500 MHz, Acetone-d_6)

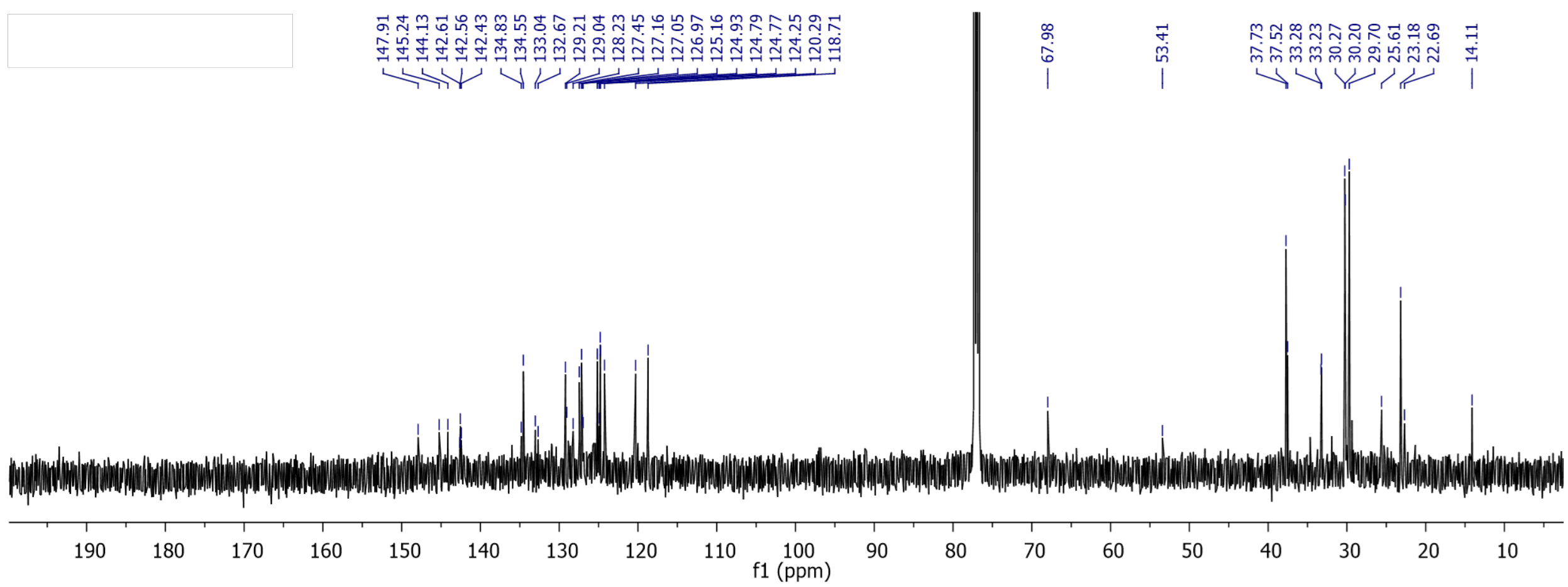

^{13}C NMR of **T2** (500 MHz, Chloroform-d)

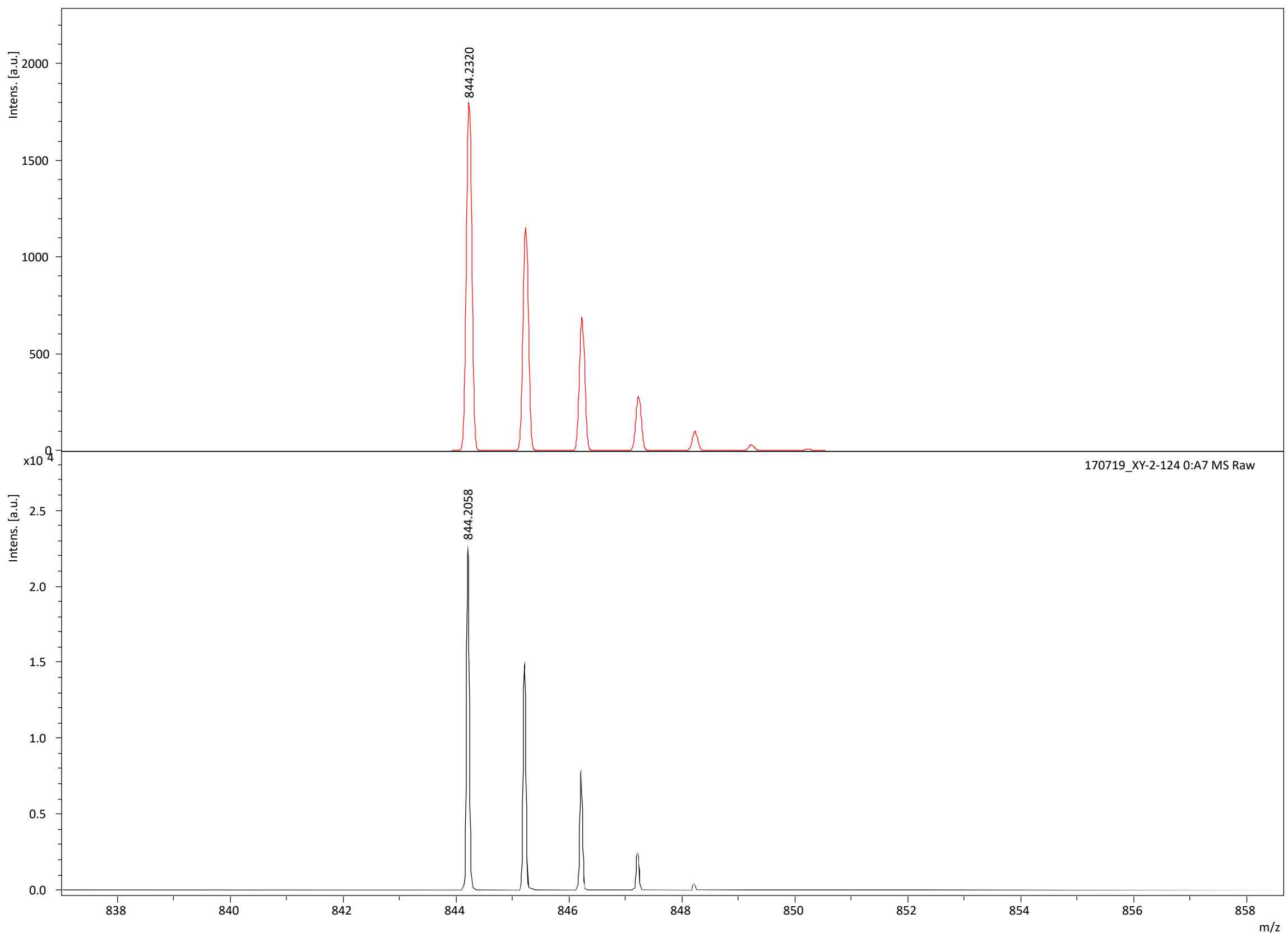

MALDI of **T2**

References

(1) Capozzi, B.; Dell, E. J.; Berkelbach, T. C.; Reichman, D. R.; Venkataraman, L.; Campos, L. M. Length-dependent conductance of oligothiophenes, *J. Am. Chem. Soc.* **2014**, *136*, 10486.

(2) Chang, W. B.; Mai, C.-K.; Kotiuga, M.; Neaton, J. B.; Bazan, G. C.; Segalman, R. A. Controlling the thermoelectric properties of thiophene-derived single-molecule junctions, *Chem. Mater.* **2014**, *26*, 7229.

(3) Chen, W.; Li, H.; Widawsky, J. R.; Appayee, C.; Venkataraman, L.; Breslow, R. Aromaticity decreases single-molecule junction conductance, *J. Am. Chem. Soc.* **2014**, *136*, 918.

(4) Nagahara, L.; Thundat, T.; Lindsay, S. Preparation and characterization of STM tips for electrochemical studies, *Rev. Sci. Instrum.* **1989**, *60*, 3128.

(5) Capozzi, B.; Xia, J.; Adak, O.; Dell, E. J.; Liu, Z.-F.; Taylor, J. C.; Neaton, J. B.; Campos, L. M.; Venkataraman, L. Single-molecule diodes with high rectification ratios through environmental control, *Nat. Nanotechnol.* **2015**, *10*, 522.

(6) Dell, E. J.; Capozzi, B.; Xia, J.; Venkataraman, L.; Campos, L. M. Molecular length dictates the nature of charge carriers in single-molecule junctions of oxidized oligothiophenes, *Nat. Chem.* **2015**, *7*, 209.

(7) Greenwald, J. E.; Cameron, J.; Findlay, N. J.; Fu, T.; Gunasekaran, S.; Skabara, P. J.; Venkataraman, L. Highly nonlinear transport across single-molecule junctions via destructive quantum interference, *Nat. Nanotechnol.* **2021**, *16*, 313.

(8) Lee, W.; Li, L.; Camarasa-Gómez, M.; Hernangómez-Pérez, D.; Roy, X.; Evers, F.; Inkpen, M. S.; Venkataraman, L. Photooxidation driven formation of Fe-Au linked ferrocene-based single-molecule junctions, *Nat. Commun.* **2024**, *15*, 1439.

(9) Li, S.; Li, J.; Yu, H.; Pudar, S.; Li, B.; Rodríguez-López, J.; Moore, J. S.; Schroeder, C. M. Characterizing intermolecular interactions in redox-active pyridinium-based molecular junctions, *J. Electroanal. Chem.* **2020**, *875*, 114070.

(10) Wang, Z. *et al.* Electrochemically controlled rectification in symmetric single-molecule junctions, *Proc. Nat. Acad. Sci. U.S.A.* **2022**, *119*, e2122183119.

(11) Bagrets, A. Spin-polarized electron transport across metal–organic molecules: a density functional theory approach, *J. Chem. Theory Comput.* **2013**, *9*, 2801.

(12) Blum, V.; Gehrke, R.; Hanke, F.; Havu, P.; Havu, V.; Ren, X.; Reuter, K.; Scheffler, M. Ab initio molecular simulations with numeric atom-centered orbitals, *Comp. Phys. Commun.* **2009**, *180*, 2175.

(13) Perdew, J. P.; Burke, K.; Ernzerhof, M. Generalized gradient approximation made simple, *Phys. Rev. Lett.* **1996**, *77*, 3865.

(14) Lenthe, E. v.; Baerends, E.-J.; Snijders, J. G. Relativistic regular two-component Hamiltonians, *J. Chem. Phys.* **1993**, *99*, 4597.
